# Potential Role of a Bistable Histidine Kinase Switch in the Asymmetric Division Cycle of *Caulobacter crescentus*

**DOI:** 10.1371/journal.pcbi.1003221

**Published:** 2013-09-12

**Authors:** Kartik Subramanian, Mark R. Paul, John J. Tyson

**Affiliations:** 1 Graduate Program in Genetics, Bioinformatics and Computational Biology, Virginia Polytechnic Institute and State University, Blacksburg, Virginia, United States of America; 2 Department of Mechanical Engineering, Virginia Polytechnic Institute and State University, Blacksburg, Virginia, United States of America; 3 Department of Biological Sciences, Virginia Polytechnic Institute and State University, Blacksburg, Virginia, United States of America; 4 Virginia Bioinformatics Institute, Virginia Polytechnic Institute and State University, Blacksburg, Virginia, United States of America; University of Illinois at Urbana-Champaign, United States of America

## Abstract

The free-living aquatic bacterium, *Caulobacter crescentus*, exhibits two different morphologies during its life cycle. The morphological change from swarmer cell to stalked cell is a result of changes of function of two bi-functional histidine kinases, PleC and CckA. Here, we describe a detailed molecular mechanism by which the function of PleC changes between phosphatase and kinase state. By mathematical modeling of our proposed molecular interactions, we derive conditions under which PleC, CckA and its response regulators exhibit bistable behavior, thus providing a scenario for robust switching between swarmer and stalked states. Our simulations are in reasonable agreement with *in vitro* and *in vivo* experimental observations of wild type and mutant phenotypes. According to our model, the kinase form of PleC is essential for the swarmer-to-stalked transition and to prevent premature development of the swarmer pole. Based on our results, we reconcile some published experimental observations and suggest novel mutants to test our predictions.

## Author Summary

Recent evidence suggests that the transition of PleC from phosphatase to kinase is induced by its own substrate, DivK. Based on experimental clues, we propose a molecular mechanism to explain this substrate-induced conformational change in PleC. The general principles of thermodynamics, enzyme-substrate reactions and the Monod-Wyman-Changeux model of allostery motivate the elementary chemical reactions proposed in our model. Formulating our hypothesis in terms of nonlinear ordinary differential equations, we show that the PleC transition could function as a bistable switch. Although initial experimental studies have suggested that the primary role of PleC is as a phosphatase, our simulations show that the PleC kinase form is relevant for the correct temporal regulation of the *Caulobacter* cell cycle.

## Introduction

The function of the cell division cycle of both prokaryotes and eukaryotes is to produce two nearly identical copies of a progenitor cell. The two progeny cells have identical genomes (modulo unavoidable mutations in the DNA replication process), and they are usually quite similar in all other aspects (called “symmetric” cell division). However, there are many cases of asymmetric cell division, in which the two progeny cells are notably different from each other [1]. An interesting example of asymmetric cell division is the freshwater bacterium, *Caulobacter crescentus*. Because *Caulobacter* populations typically live in low-nutrient environments, they have developed a strategy of asymmetric cell division to limit intraspecific competition [2]. During the cell division process, proteins are unequally distributed to the two halves of the cell, giving rise to two morphologically distinct daughter cells. One daughter cell (the stalked cell) is anchored to its place of birth via an appendage called the stalk, while the other daughter cell (the swarmer cell) is equipped with a flagellum and pilus that allows it to swim away from its place of birth. Hence, even though the total number of cells doubles, the number of stalked cells at a specific location stays the same. Another key difference is that, after cell division, the stalked cell immediately initiates a new round of DNA replication and cell division, while the wandering swarmer cell is not competent for DNA replication (it is in a prokaryote version of G1 phase). Once the swarmer cell finds a nutritionally suitable location, it will differentiate into an immobile stalked cell, initiate DNA replication, and establish a new population.

**Figure 1:**
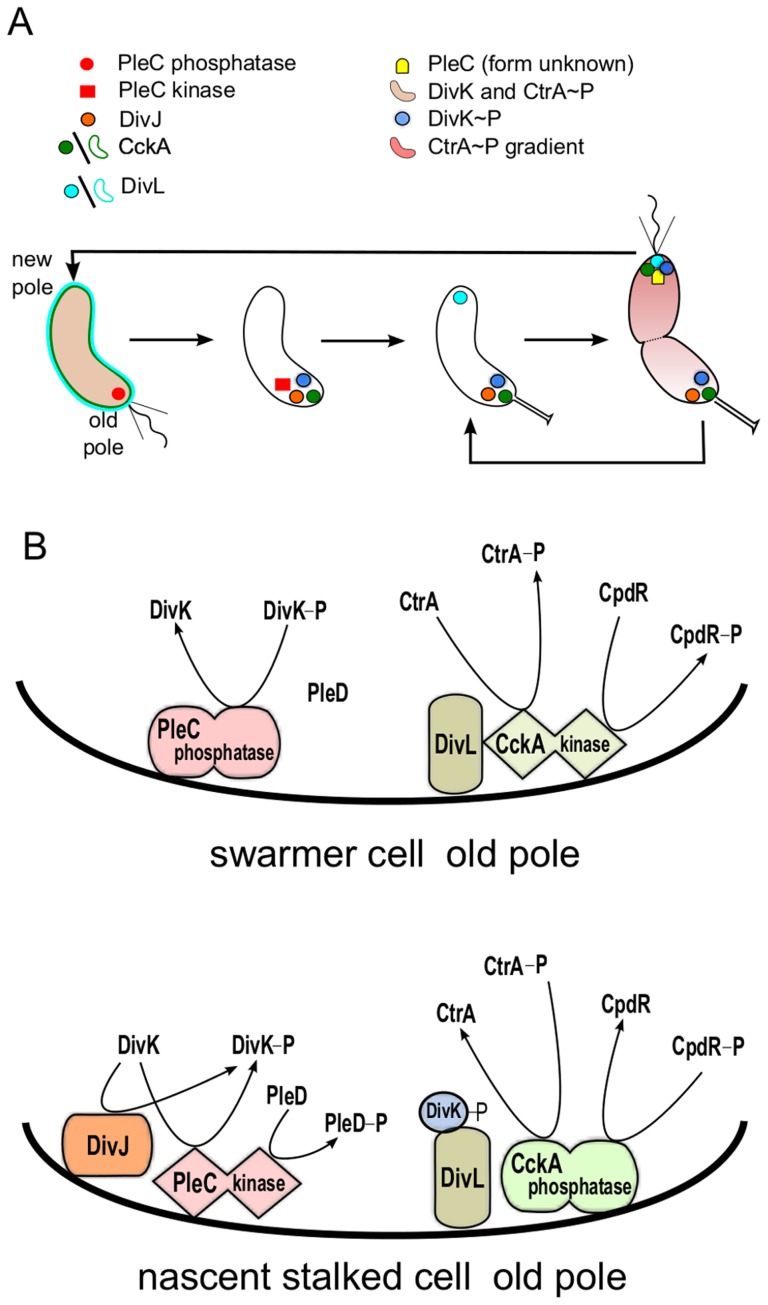
Morphological transitions in *Caulobacter crescentus* are governed by changes in localization and activity of proteins. (**A**) Schematic representation of the *Caulobacter crescentus* cell cycle. The cell undergoes a series of morphological changes from swarmer cell (left)→nascent stalked cell→stalked cell→pre-divisonal cell (right). These events are driven by changes in the activity and localization of cell cycle proteins. In particular, notice that DivL (light blue) and CckA (green) are uniformly distributed on the membrane in the swarmer cell but localized at the poles in the stalked cell. (**B**) Model of the status of PleC and CckA activity at the old pole in the swarmer cell and in the nascent stalked cell. In the swarmer cell, DivJ is not localized or activated. As a result, PleC is a phosphatase and CckA is a kinase. In the stalked cell, DivJ is localized to the old pole, causing PleC to flip to the kinase form, which in turn induces CckA to switch to a phosphatase.

Orchestration of this asymmetric cell division cycle requires proper temporal and spatial regulation of several key proteins (see **Figure 1A**). The temporal dynamics of these proteins was captured in a pair of papers by Li *et al.* [3], [4]. At least two of these proteins, PleC and CckA, are bifunctional, capable of acting as either phosphatase or kinase. PleC kinase activity is up-regulated by its own response regulator, DivK. It is unknown how DivK alters the activity of its own phosphorylating enzyme, PleC. DivK is present at roughly constant level throughout the cell cycle [5]. However, PleC is a phosphatase during the swarmer stage of the cell cycle and kinase during the stalked stage (see **Figure 1B**). It would be interesting to know how this cross-talk between PleC-kinase and its substrate, DivK, is restricted to the stalked stage of the cell cycle.

At the level of physiology, whether a cell has a stalk or a flagellum depends on the phosphorylation status of the proteins DivK, PleD and CtrA. In the swarmer cell, CtrA∼P (the active, phosphorylated form of CtrA) binds to the origin of replication on the *Caulobacter* chromosome and inhibits initiation of DNA replication [6]. During the transition from swarmer to stalked cell, CtrA gets dephosphorylated and degraded, thereby lifting the block on DNA replication. In addition, CtrA affects the transcription of over 125 genes, so periodic changes in CtrA activity causes widespread changes in the expression profile of *Caulobacter* genes during the cell division cycle [7], [8]. DivK, on the other hand, is unphosphorylated in the swarmer cell and gets phosphorylated during the transition to the stalked cell. In the phosphorylated state, DivK initiates a pathway for stalk formation [9]. It is also responsible (indirectly) for the dephosphorylation and proteolysis of CtrA [10]–[12].

The phosphorylation states of DivK and CtrA are governed by the bifunctional histidine kinases PleC and CckA, respectively. Both PleC and CckA can switch between two conformations: a kinase conformation and a phosphatase conformation [9], [11] (see **Figure 1B**). Typically, in bacteria the change in activity of a bifunctional histidine kinase is brought about by an external signal molecule binding to the sensor region of the protein [13]. However, the change in PleC from a phosphatase to a kinase is brought about by its substrate, DivK [9]. In fact, the sensor domain of PleC is not essential for its function [14]. This interaction, where substrate binding to a bifunctional histidine kinase changes its function, has, to our knowledge, been observed only for PleC in *Caulobacter*. It has been suggested that DivK up-regulates PleC kinase activity preferentially in stalked cells because it is in stalked cells where DivK∼P and PleC are co-localized at the poles [9].

The initial phosphorylation of DivK during the swarmer-to-stalked transition is brought about by a kinase DivJ that localizes to the old pole. Hence, DivJ is considered as the enzyme that initiates the swarmer-to-stalked transition [15], [16]. A second and perhaps more crucial function of PleC kinase is to phosphorylate PleD, a diguanylate cyclase enzyme. On getting phosphorylated, PleD monomers dimerize and localize to the cell pole [17]. Active PleD converts two molecules of GTP into cyclic di-GMP, which signals production of the stalk [9]. Although mutations in *divJ* and *pleC* are not lethal, they result in growth and morphological defects in the cell. *pleC*::Tn*5* mutants are stalkless [18], [19], while *divJ*-null mutants are filamentous and have elevated levels of CtrA-dependent transcription products [20], [21]. DivK∼P level is elevated in *pleC*::Tn*5* mutants and reduced in Δ*divJ* background. Δ*divJ pleC*::Tn*5* double mutants exhibit an even lower level of DivK∼P than Δ*divJ* single mutants [21], indicating that PleC has a partial role, at least, as a DivK kinase.

CckA acts as a kinase in the swarmer cell, keeping the level of CtrA∼P high, which in turn blocks DNA replication [22]. In the stalked cell, CckA becomes a phosphatase, and CtrA gets dephosphorylated, allowing initiation of DNA replication [23]. DivL, a tyrosine kinase has been implicated in maintaining CckA in the kinase state [11], [12], [24], [25]. DivL can phosphorylate CtrA *in vitro* [18]. However, *in vivo* its role in maintaining a high level of CtrA∼P is indirect [24]. Multiple lines of evidence support the idea that DivL promotes CtrA phosphorylation via activation of CckA kinase. (a) *divL* mutants show marked reduction not only in CtrA∼P but also in CckA∼P [24], [26] and CpdR∼P [26]. (b) The phenotype of *divJ* over-expression mutants is alleviated by mutations in *divL* [20]. (c) DivK∼P is known to bind to DivL and interfere with its ability to activate CckA kinase [11]. Although the mechanism by which DivL influences CckA is unclear, DivL seems to be the intermediate by which the PleC-DivJ-DivK∼P axis regulates the level of CtrA∼P.

CckA's second substrate, CpdR, is phosphorylated and inactive in swarmer cells [23]. When CckA becomes a phosphatase in the stalked cell, active CpdR turns on the ClpXP proteolytic machinery for degrading CtrA [27], [28]. In this manner, CckA governs both dephosphorylation and proteolysis of CtrA.

**Figure 2:**
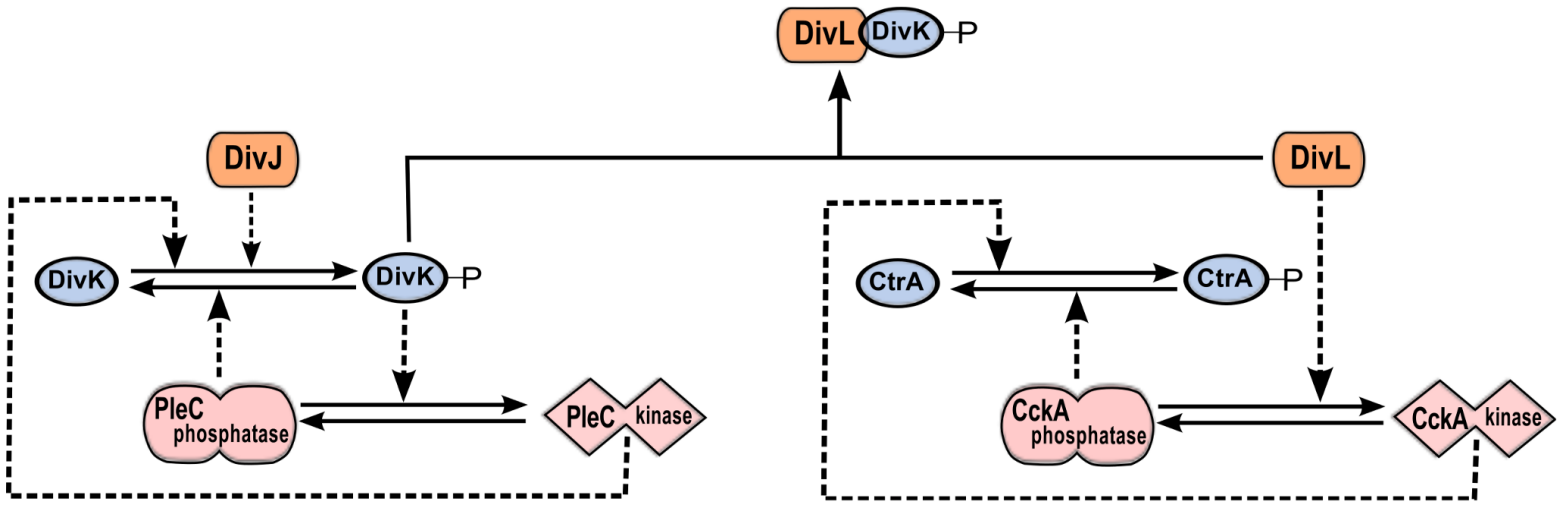
The DivJ-PleC-DivK and DivL-CckA-CtrA modules are coupled via DivK∼P-DivL interaction. PleC kinase and DivK∼P are involved in a positive feedback loop. By phosphorylating DivK, DivJ biases the positive feedback loop toward the PleC kinase state. DivL up-regulates the kinase form of CckA, which in turn phosphorylates CtrA. The phosphorylated form of DivK∼P binds to and inactivates DivL, causing CckA to revert to the phosphatase form and dephosphorylate CtrA.

Taken together, these observations suggest that PleC-DivJ-DivK and DivL-CckA-CtrA are crucial drivers of the swarmer-to-stalked transition, as summarized in **Figure 1** and **Figure 2**.

Here, we propose a mechanism for ligand-dependent modifications of the bifunctional histidine kinase, PleC. The mechanism consists of elementary chemical reactions describing ligands (either DivK or DivK∼P) binding to the histidine kinase dimer in either its phosphatase or kinase form. The binding states determine the rates of the autophosphorylation, phosphotransfer, and phosphatase reactions catalyzed by PleC. If DivK∼P is more efficient than unphosphorylated DivK at promoting the transition of PleC from phosphatase to kinase, then PleC and DivK∼P would be involved in a positive feedback loop. Such positive feedback loops are well-known for their tendency to function as bistable toggle switches [29], and toggle switches are well-known for their roles in cellular decision-making [30]–[32] including critical transitions in the eukaryotic cell cycle [33]–[35].

In the Supplementary Material (**Text S1**), we show that a detailed model of the interactions between DivK and PleC, under reasonable conditions on the rate constants (or propensities) of these reactions, exhibits robust bistability as a function of DivJ activity. That is, by carrying out the initial, limited phosphorylation of DivK, DivJ can function as the “toggle bar” for flipping the bistable switch from the PleC-phosphatase state to the PleC-kinase state. When DivJ activity is low (swarmer cell), PleC is a phosphatase and DivK is predominantly dephosphorylated. As DivJ activity rises, enough DivK gets phosphorylated to flip the PleC switch to the kinase form (stalked cell). By coupling DivK∼P to DivL, we show that the PleC switch can induce the transition of CckA from kinase to phosphatase form, causing CtrA∼P and CpdR∼P levels to drop in the nascent stalked cell (**Figure 1B**).

This model of a PleC bistable switch is an intermediate step on the way to a full spatial model of the asymmetric division cycle in *Caulobacter* cells (in preparation). Using a model based on ordinary differential equations (biochemical kinetics of spatially homogeneous reactions), we address in this paper only certain features of the control system that are independent of the complex spatio-temporal choreography of the cell cycle control system. In particular, we validate our model of the PleC switch against known mutant phenotypes, and then we discuss some predictions of the model: (a) over-expressing DivK should result in a loss of asymmetry and cell cycle arrest in the stalked cell stage, (b) PleC kinase is required to ensure that the nascent swarmer pole will mature only after cytokinesis, and (c) the swarmer-to-stalked transition is robust to fluctuations in nutrients available in the environment.

## Results

### Proposed mechanism of substrate-induced conformational changes in PleC

**Figure 3:**
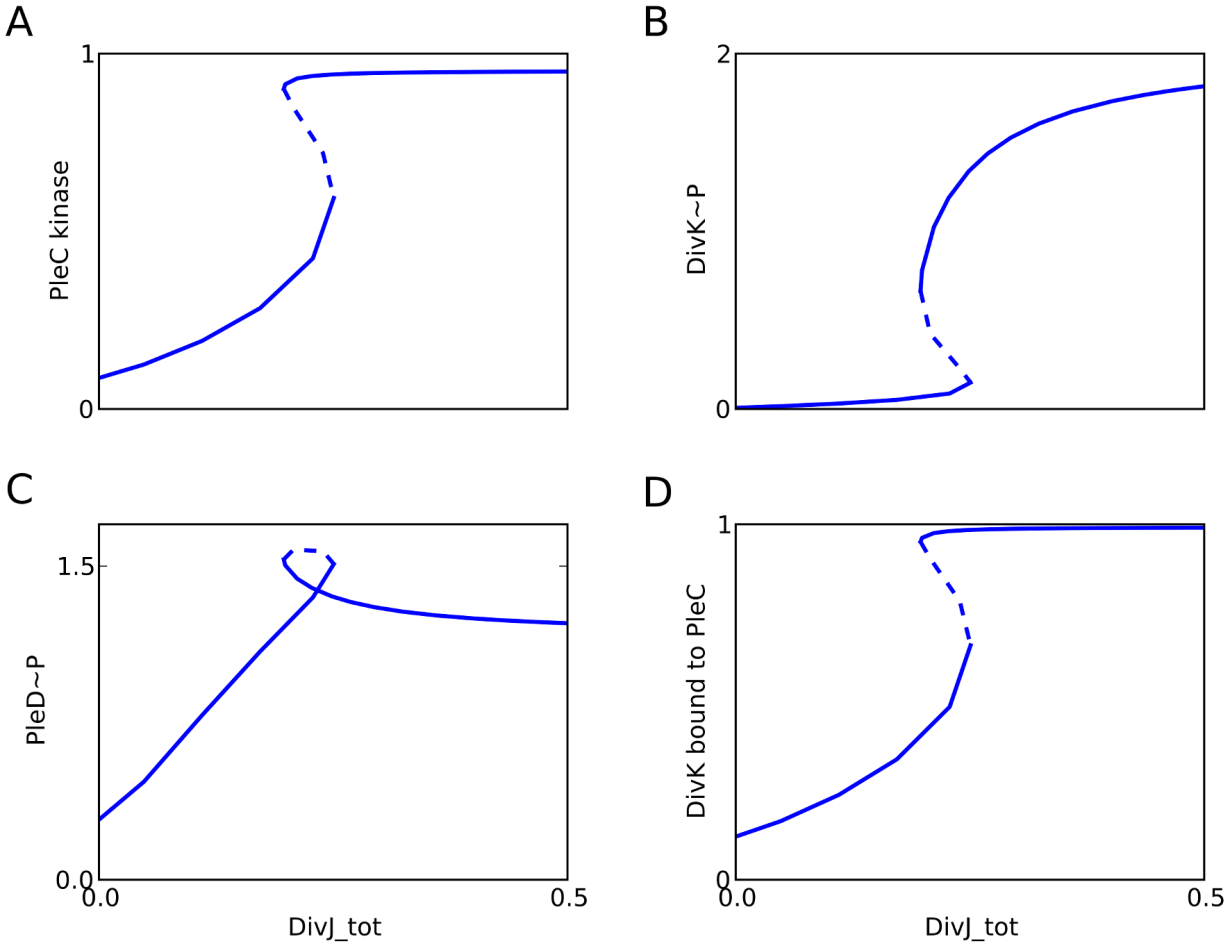
DivJ initiates the PleC phosphatase-to-kinase transition. For the full model (**Figure S1**), we plot signal-response curves (one-parameter bifurcation diagrams) for the steady state levels of (**A**) PleC kinase, (**B**) DivK∼P, (**C**) PleD∼P, and (**D**) DivK bound to PleC as functions of total DivJ (the parameter DivJ_tot in the model). Solid lines, stable steady states; dashed lines, unstable steady states. In our model, PleC_tot = constant = 1.0, but total DivK, total CtrA, and total PleD depends on DivJ_tot (see **Figure S6**).

Our detailed mechanism of substrate-induced conformational changes in PleC is presented in the Supplementary Material (**Text S1**). The model is based on the following considerations. PleC is a homodimeric, bifunctional histidine kinase. It can bind to either DivK or DivK∼P. As a kinase, it phosphorylates DivK to DivK∼P, and as a phosphatase it hydrolyzes DivK∼P back to DivK. We assume that, when DivK or DivK∼P are bound to both subunits of PleC, the enzyme undergoes a concerted conformational change from its phosphatase form to its kinase form. The conformational change is described in the manner of the Monod-Wyman-Changeux [36] theory of allosteric enzymes. A detailed model of PleC-DivJ-DivK-PleD interactions contains 38 biochemical species (**Table S4**, Eq. 1–38; **Figure S1A** and **B**), many of which are involved in null-cycles. To build a kinetic model of this reaction network, we must assign reasonable values to all the forward and reverse rate constants (*k*_f_ and *k*_r_), respecting the fact that *k*_f_/*k*_r_ = *K*_eq_ = exp(−Δ*G* ^0^/*RT*), where Δ*G* ^0^ is the standard Gibbs free energy change and *K*_eq_ is the equilibrium constant for the reaction. In the Supplementary Material **(Text S1)** we assign reasonable Δ*G* ^0^ values to every reaction in the network, and then assign *k*_f_ and *k*_r_ values consistent with the computed equilibrium constants. In this way, we are assured that our kinetic model satisfies the Principle of Detailed Balance around all null-cycles. (For a null cycle, Δ*G* ^0^ = 0 and *K*_eq_ = 1; hence, the product of forward rate constants around the cycle = the product of reverse rate constants around the cycle.) Having built a kinetic model that is consistent with the thermodynamic requirements of the histidine kinase (PleC)—response regulator (DivK) system, we then show (see **Figure 3**) that the ‘two component’ system does indeed exhibit bistability as a function of DivJ activity.

In the next subsections, we examine biochemically relevant features of this bistable control system.

### DivJ-dependent phosphorylation of DivK is crucial for switching PleC from a phosphatase to a kinase

*ΔdivJ* cells are filamentous [20], [21], show mislocalized stalks and delocalized DivK [5]. In addition, the level of phosphorylation of DivK in *ΔdivJ* cells is reported to be only 44% of wild-type level [21]. Not surprisingly, CtrA∼P level is higher in this deletion mutant [20]. Furthermore, mutations in *divJ* have an adverse effect on cell division rate [20], [37], [38]. Hence, DivJ is considered to be a cell-fate determinant, essential for a smooth swarmer-to-stalked transition [39].

Paul *et al.* [9] suggested that DivJ initiates the PleC phosphatase-to-kinase transition, by a positive feedback loop: DivK, on being phosphorylated by DivJ, activates PleC autokinase, and PleC kinase makes more DivK∼P. Their experiments, however, indicate that PleC kinase activity is up-regulated by DivK irrespective of DivK's phosphorylation state. Given that the total concentration of DivK remains the same throughout the cell cycle [5], why isn't PleC a kinase at all times?

Presumably, the phosphatase form of PleC has a higher affinity for its substrate DivK∼P than for its product DivK. Therefore, even though the PleC phosphatase-to-kinase transition may be promoted by either DivK∼P or DivK, DivK∼P has a greater propensity than DivK to induce the conformational change. Once PleC becomes a kinase, it produces more DivK∼P, which enhances the rate of change from phosphatase to kinase. This self-reinforcing positive feedback loop between DivK∼P and PleC kinase can turn the PleC transition into a bistable “toggle” switch [29].

As shown in **Figure 3A**, DivJ can function as the lever of this toggle switch. As the activity of DivJ increases, PleC switches abruptly from a steady state of low kinase activity to a steady state of high kinase activity. DivK also transitions from a mostly-unphosphorylated steady state to a mostly-phosphorylated steady state (**Figure 3B** and **Figure S6B**), as does PleD as well (**Figure 3C** and **Figure S6F**). We propose that this toggle switch underlies the swarmer-to-stalked transition, where the arrival of DivJ at the old pole triggers PleC to switch to its kinase form, thereby triggering a new stalk end through PleD phosphorylation. It has been shown that upon glucose starvation, DivJ localization is inhibited, and the proportion of swarmer cells in the population doubles [39]. To test the signal-response curves in our model, it would be interesting to see if single cells can toggle between swarmer and stalked morphology upon changing nutrient composition.

According to Paul *et al.*, accumulation of DivK∼P at the poles causes its local concentration to increase beyond a threshold required for the activation of PleC kinase. Our model does not address this possibility because (at present) it does not take space into account. While we cannot rule out the contribution of polar localization, our model shows that it is not essential for the phosphatase-to-kinase transition. Our simulations indicate that a large fraction of PleC kinase is bound to DivK (**Figure 3D**). Hence, it is possible that localization of DivK∼P is not the cause but the consequence of PleC kinase up-regulation. PleC kinase molecules may serve as docking sites for DivK molecules at the flagellar pole. PleC phosphatase on the other hand need not have any bound DivK. This picture is in agreement with observations that PleC, DivJ and DivL contribute to localization of DivK∼P to the poles [19], [40].

### Over-expressing DivK may block the cell cycle in the stalked cell stage

*In vitro* experiments show that PleC kinase activity increases in response to increasing DivK concentration, even in the absence of DivJ [9]. The specific activity of PleD in forming cyclic di-GMP was used as a proxy to measure PleC kinase activity. Surprisingly, the specific activity of PleC kinase *in vitro* is two-fold greater in the presence of DivK_D53N_, a mutant form of DivK that does not get phosphorylated. This indicates that DivK need not be phosphorylated to induce a conformational change in PleC. *In vivo*, however, PleC remains a phosphatase in the DivK-rich swarmer cell. Another odd result of the assay is that the specific activity of PleC kinase drops sharply at high DivK concentrations.

**Figure 4:**
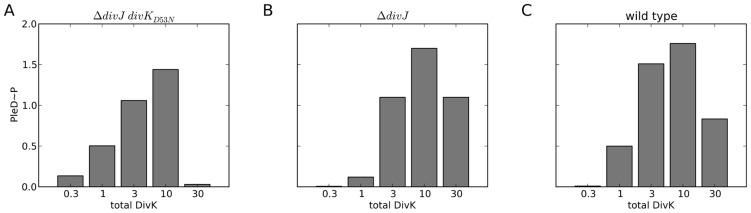
Over-expressing DivK causes a drop in PleD phosphorylation. The steady state level of PleD∼P is plotted against increasing amount of total DivK for (**A**) *ΔdivJ divK_D53N_*, (**B**) *ΔdivJ*, and (**C**) wild type background. Although the absolute levels vary among the three cell types, in each case PleD∼P level shows an initial increase followed by a drop at high DivK.

To reproduce these results in Δ*divJ* mutants, we set [DivJ] = 0 in our simulations (**Table S8**). To simulate the *divK_D53N_* mutation, we set the rates of all phosphotransfer reactions to zero (**Table S8**). In **Figure 4** we plot steady-state PleD phosphorylation level against increasing total concentration of DivK (from 0.3 to 30). Our simulations show a qualitative similarity to the experiments [9]. PleD∼P level rises at first and then drops at high [DivK] (**Figure 4A–C**). PleD∼P levels in Δ*divJ divK_D53N_* simulations (**Figure 4A**) are comparable to PleD∼P levels in Δ*divJ* (**Figure 4B**) and wild-type (**Figure 4C**) simulations. These results support the findings by Paul *et al.* [9] that unphosphorylated DivK is also able to up-regulate PleC kinase. There is a sharp drop in PleD phosphorylation at high [DivK] because PleC shifts predominantly to DivK-bound forms that do not have a free binding site for PleD (**Figure 3D**) and therefore cannot phosphorylate it. Product inhibition by cyclic di-GMP may also play a significant role [41], but this effect is not included in our model.

**Figure 5:**
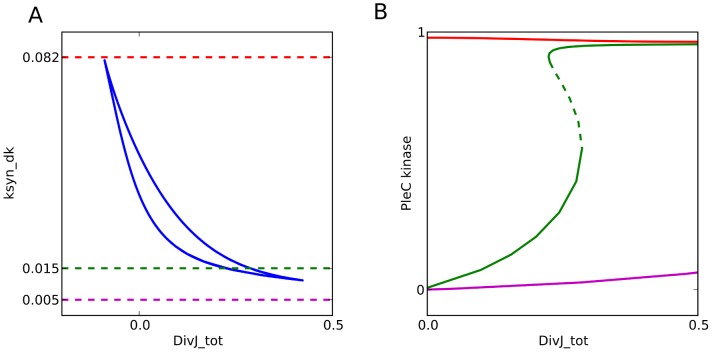
Over-expressing DivK causes activation the PleC switch independent of DivJ. (**A**) Two-parameter bifurcation diagram, indicating how the PleC switch behaves in cells expressing different levels of DivJ_tot and DivK_total (*k*_syn_dk_ is the rate constant for synthesis of DivK). PleC exhibits bistability within the crescent-shaped region bounded by the blue lines. (**B**) One-parameter bifurcation diagrams (signal-response curves) for three different values of *k*_syn_dk_, indicated by the dashed horizontal lines in panel A. Notice that PleC kinase level is always low if *k*_syn_dk_<0.011 and always high if *k*_syn_dk_>0.082.

Since DivK is capable of activating PleC kinase in the absence of DivJ, we plotted a two-parameter bifurcation diagram to estimate the effect of varying concentrations of DivJ and DivK on PleC activity (**Figure 5A**). The enclosed bistable region tapers off as we increase either total DivJ or total DivK (*k*_syndk_). This implies that at moderate concentrations of DivK (e.g., *k*_syndk_ = 0.015), the PleC phosphatase-to-kinase transition is robust and dependent on the activity and localization of DivJ (**Figure 5B**). However, increasing DivK in the cell would lead to transitions that are less robust and independent of DivJ. We predict that a 5- to 10-fold increase in DivK concentration will result in PleC being locked in the kinase form, and the cell will be blocked in the stalked stage of the cell cycle. We propose that *in vivo* the total concentration of DivK is low enough that it needs to be phosphorylated in order to induce PleC to become a kinase. In this case, the bistable PleC switch becomes reliant on the appearance of DivJ activity rather than on the polar accumulation of DivK.

### The PleC-DivJ-DivK switch confers bistability to the DivL-CckA-CtrA module

The DivL-CckA-CtrA module bears a striking resemblance to DivJ-PleC-DivK switch. Nonetheless, there are important differences. DivL can phosphorylate CtrA *in vitro*, but this reaction is of no significance *in vivo* [22], [42]. Unlike PleC, which directly transfers its phosphoryl group to an aspartate residue on DivK, CckA relies on a series of phospho-transfer events [23]. To this end, it has an additional aspartate-containing domain which first picks up the phosphoryl group from the histidine residue and passes it on to the histidine residue of a downstream histidine phosphotransfer (HPt) protein called ChpT [43]. Finally, ChpT relays the phosphoryl group to the aspartate residue on the response regulator CtrA. In our mathematical equations, we model ChpT and CckA as a single protein, CckA, whose transition from phosphatase to kinase is promoted by binding to substrate, CtrA. The third difference is that CtrA is not known to up-regulate CckA kinase, so there is no reason to expect bistability in the CckA-ChpT-CtrA phospho-relay system.

**Figure 6:**
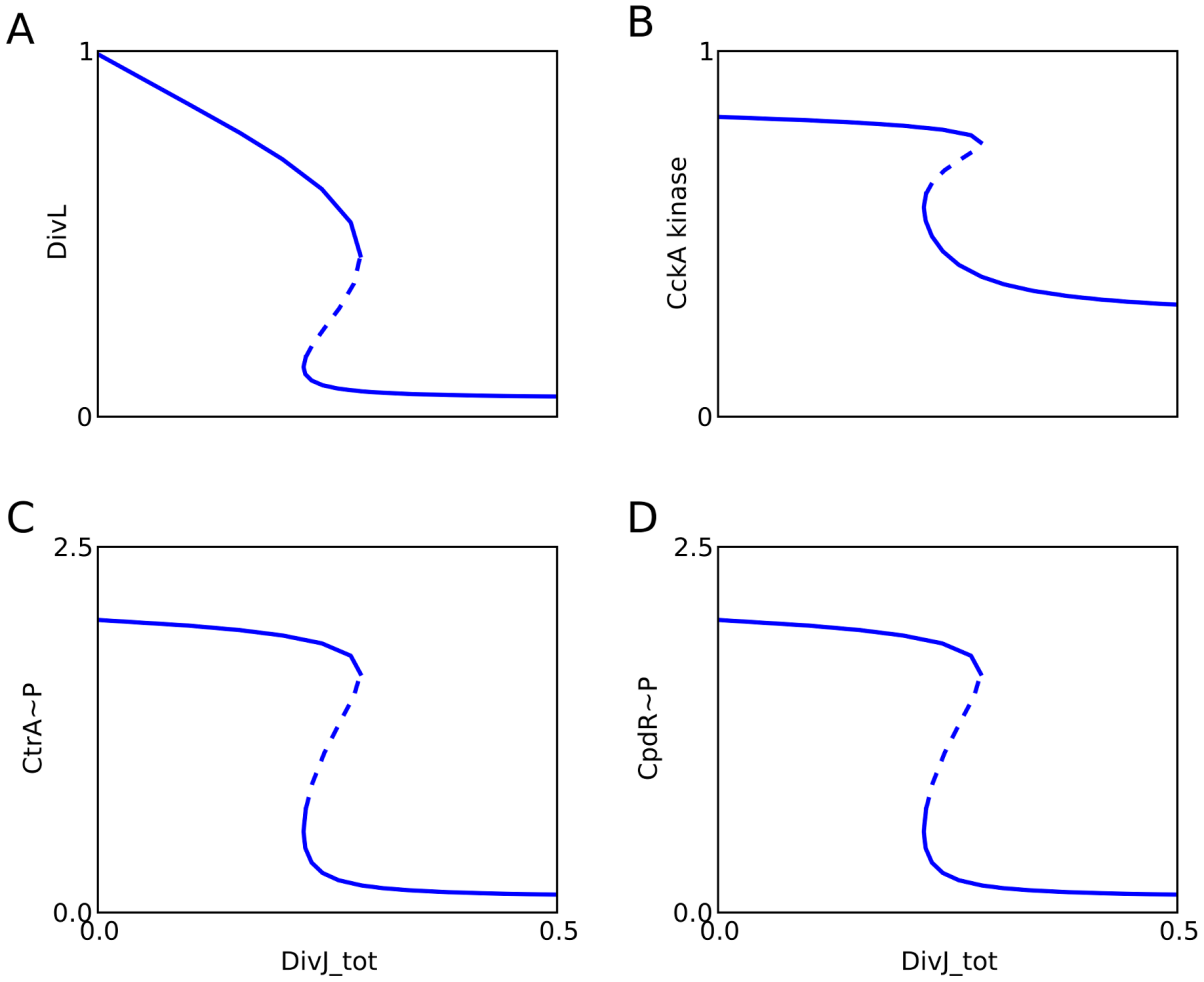
The DivJ-PleC-DivK module controls the DivL-CckA-CtrA module. When the PleC switch is activated, DivK∼P binds to DivL and inactivates components of the CckA module. One parameter bifurcation diagrams show the steady state levels of (**A**) DivL, (**B**) CckA kinase, (**C**) CtrA∼P, and (**D**) CpdR∼P as functions of DivJ_tot.

It is a well-established fact that DivK∼P inhibits CtrA activity, and the mechanistic details of this process have become progressively clear. Initial experiments showed that DivK∼P down-regulates CckA kinase activity [12]. Later experiments indicated that DivL is required for maintaining CckA as a kinase, and that DivK∼P binding to DivL inhibits this effect [11], [44]. Since the mechanistic details regarding how DivL influences CckA activity are currently unknown, we model this process phenomenologically, using a Hill function to describe how DivL promotes CckA kinase. We couple the PleC-DivK∼P bistable switch to the CckA kinase-to-phosphatase transition by having DivK∼P bind to and inactivate DivL. In the swarmer cell, DivJ is absent and the PleC switch is in the phosphatase state (DivK unphosphorylated). Hence, DivL is active and maintains CckA in the kinase state (CtrA phosphorylated). The up-regulation of DivJ is the trigger for the swarmer-to-stalked transition. DivJ activity flips the PleC switch to the kinase state, DivK gets phosphorylated and binds to DivL. DivL activity drops abruptly (**Figure 6A**), and consequently CckA returns to its default phosphatase form (**Figure 6B**). As a result, CtrA becomes dephosphorylated and inactive (**Figure 6C** and **Figure S6D**), and CpdR becomes dephosphorylated and active (**Figure 6D**).

**Figure 7:**
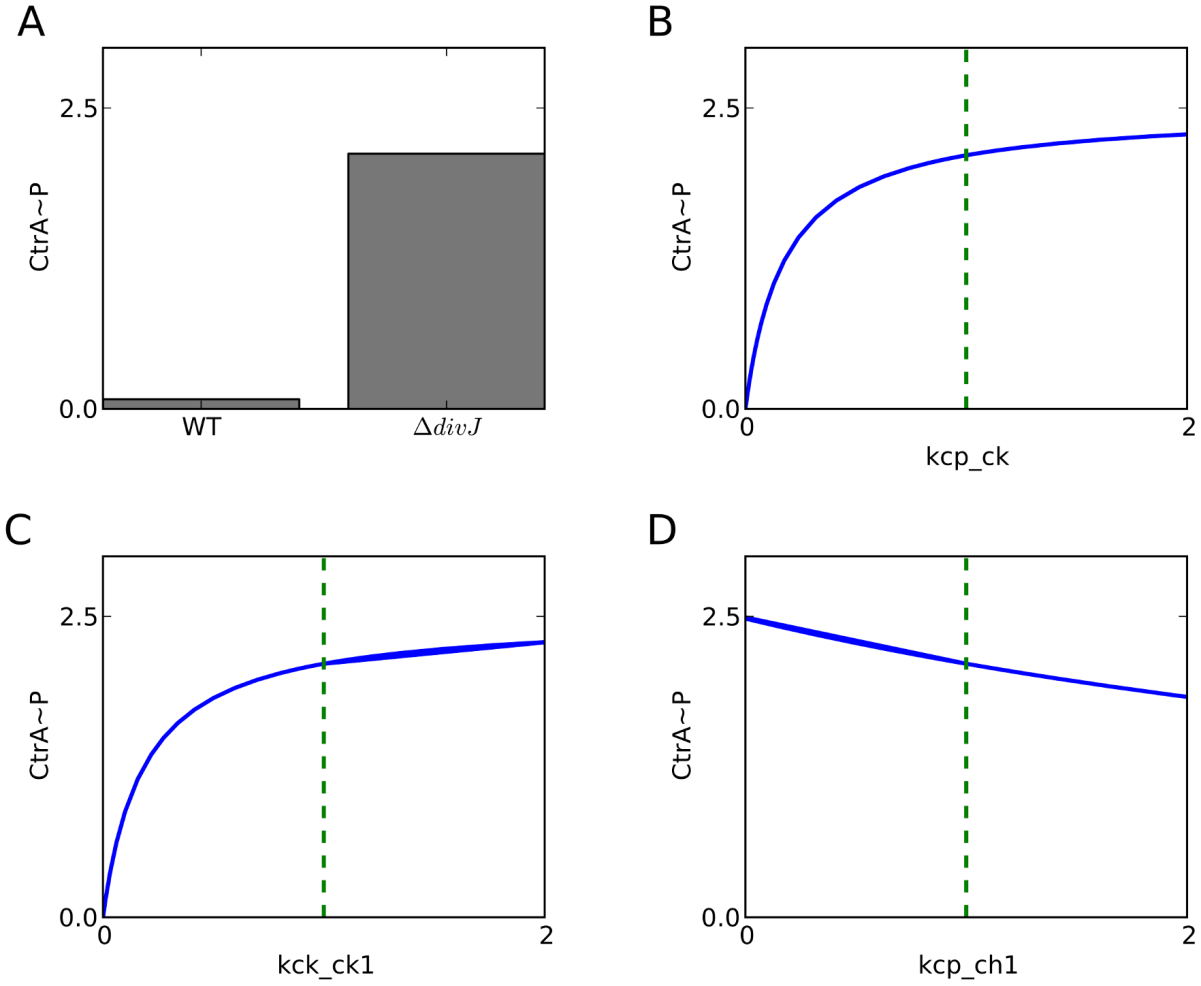
Point mutations in CckA suppress the *ΔdivJ* mutant phenotype by affecting CckA kinase activity. (**A**) *ΔdivJ* cells show higher CtrA∼P level than their wild type counterpart. Point mutations in CckA and DivL are known to suppress the mutant phenotype. To understand how, we plot the steady-state level of CtrA∼P as a function of rate constants governing (**B**) DivL activating CckA kinase (*k*_cp-ck_), (**C**) CckA kinase activity (*k*_ck-ck1_), and (**D**) CckA phosphatase activity (*k*_cp-ch1_). The vertical green lines indicate the values of the rate constants in the *ΔdivJ* background. Reducing the value of *k*_cp-ck_ or *k*_ck_ck1_ causes a corresponding reduction in CtrA∼P level. However, increasing *k*_cp_ch1_ does not cause CtrA∼P to fall to its wild-type level.

The proposed coupling of these switches is supported by experimental evidence that a Δ*divJ* mutant can be rescued by point mutations in *divL* and *cckA* genes [20]. CtrA activity, which is high in Δ*divJ* cells (**Figure 7A**), is restored to normalcy by point mutations in *divL* and *cckA* that interfere with CtrA phosphorylation (**Figure 7B–D**). As expected, CtrA∼P level in a Δ*divJ* mutant can be reduced by decreasing the specific activity of DivL (**Figure 7B**). Interestingly, our simulations show that decreasing the specific activity of CckA kinase lowers the level of CtrA∼P (**Figure 7C**), but increasing the specific activity of CckA phosphatase does not restore CtrA∼P level (**Figure 7D**). Hence, we predict that the point mutations in CckA that rescue Δ*divJ* mutants do so by reducing the kinase activity of CckA.

### Simulations of *divK_D90G_* are consistent with observed phenotypes

To simulate the consequences of the *divK_D90G_* mutation, we make note of the fact that, *in vitro*, autophosphorylation of PleC is markedly reduced in the presence of DivK_D90G_ [9]. This fact indicates that DivK_D90G_, unlike its wild-type counterpart, is unable to up-regulate the kinase form of PleC. Since DivK_D90G_ is not an allosteric ligand, we set 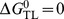, and accordingly updated the equilibrium constants and parameters for all the concerned reactions (**Table S8**). In addition, although DivK_D90G_ is phosphorylated to the same extent as wild type DivK, it is unable to bind to DivL [11]. Hence, we altered the binding equilibrium of DivK_D90G_ to DivL (**Table S8**).

**Figure 8:**
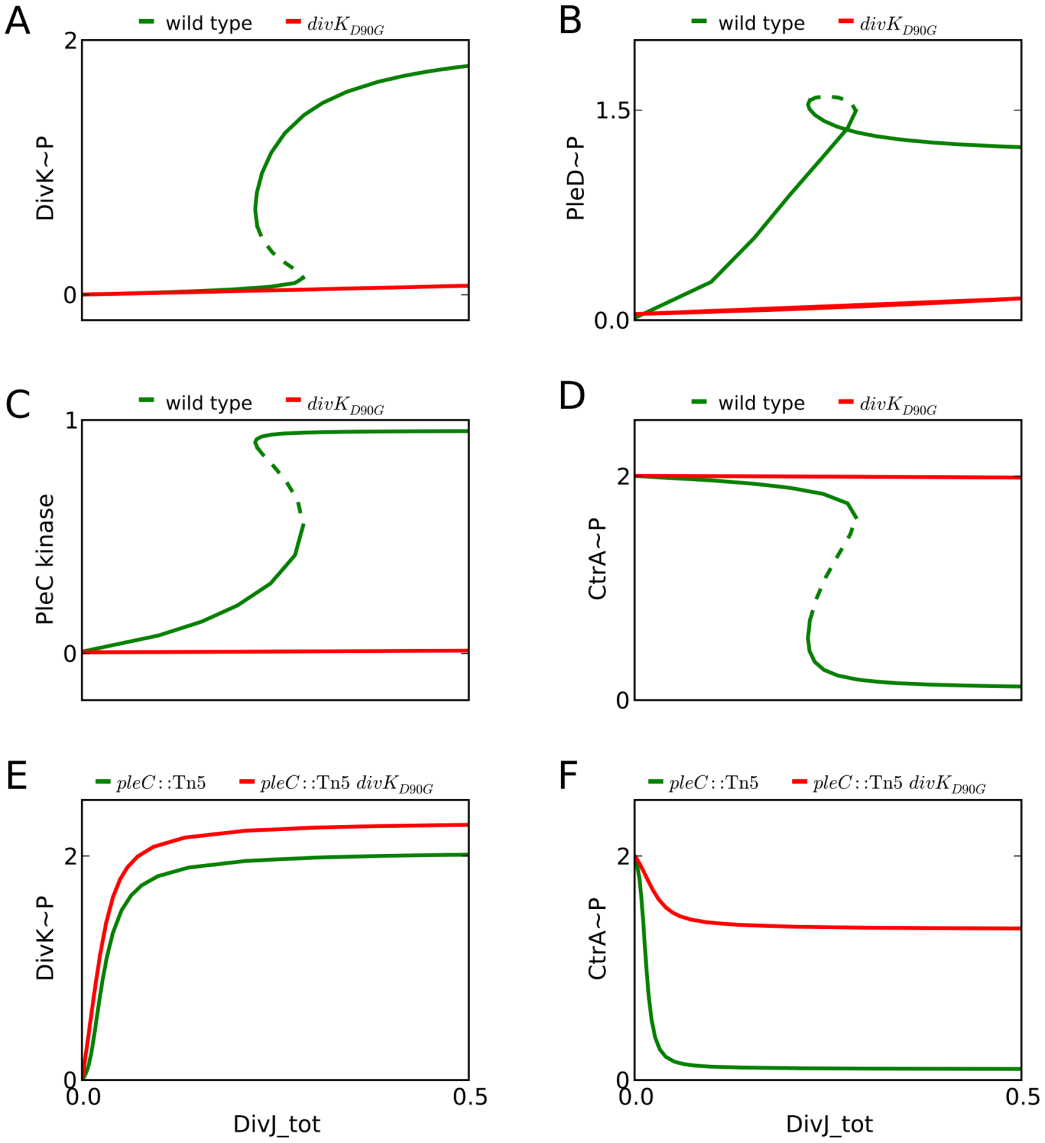
Simulations capture the physiological effects of *divK*_D90G_ mutants. *divK_D90G_* mutants are unable to activate PleC kinase or bind to and inactivate DivL. One parameter bifurcation diagrams compare the wild type (green line) and mutant level (red line) of (**A**) DivK∼P, (**B**) PleD∼P, (**C**) PleC kinase, and (**D**) CtrA∼P. *divK*_D90G_ mutants also suppress the effects of *pleC*:Tn*5* mutants. Our simulations show that (**E**) the steady state levels of DivK∼P are the same in *pleC*::Tn*5* (green line) mutant cells and in *pleC*:Tn*5 divK*_D90G_ double mutants (red line). However, (**F**) CtrA∼P is restored to wild-type level in the double mutant.

Using the altered parameter set, we tried to reproduce two known phenotypes of *divK_D90G_* cells. Filamentous *divK_D90G_* cells initiate swarmer progeny-specific development (SPD) prematurely. SPD defines a range of cell cycle events, including activation of the flagellum, development of pili, release of the flagellum and ultimately development of the stalk [45]. It is important that these events take place in a timely manner and that they are restricted to the newborn swarmer cell. Filamentous *divK_D90G_* mutants, however, initiate SPD in the pre-divisional cell. In particular, pilin synthesis (a part of SPD) requires CtrA∼P. Hence, we examined whether CtrA∼P level is increased in simulations of *divK_D90G_* mutant cells. **Figure 8** compares one-parameter bifurcation diagrams for wild-type (green) and mutant (red) cells. The levels of DivK∼P (**Figure 8A**), PleD∼P (**Figure 8B**) and PleC kinase (**Figure 8C**) are much lower in mutant cells, while CtrA∼P level remains high (**Figure 8D**). This could potentially lead to initiation of SPD.

The *divK_D90G_* mutation is a suppressor of the *pleC*::Tn*5* mutant phenotype. Cells lacking PleC show extended periods of bipolar localization of DivK∼P and also fail to develop stalks. A *pleC*::Tn*5 divK_D90G_* double mutant does not show any of these defects [45]. Our simulations show that DivK∼P level increases and CtrA∼P level drops in *pleC*::Tn*5* background (**Figure 8E and F**). Since DivK remains phosphorylated in the absence of PleC, it is not dislodged from the poles [19]. DivK∼P binds to DivL and suppresses CtrA phosphorylation (**Figure 8F**), thus preventing SPD. However, in the *pleC*::Tn*5 divK_D90G_* double mutant, CtrA∼P level remains high in spite of elevated DivK∼P (**Figure 8E–F**, red line). This result is in accordance with the finding that CckA∼P, CtrA∼P and Cpdr∼P levels are high when the binding of DivK∼P to DivL is weakened [11]. Restoration of CtrA∼P in the double mutant allows flagellar pole development. Hence, the restoration of unipolar localization of DivK in *pleC*::Tn*5 divK_D90G_* double mutant may be a natural consequence of the inability of DivK_D90G_ to bind to DivL.

### The PleC kinase activity may prevent premature swarmer progeny-specific development

Although PleC is bifunctional, its designation in the cell has primarily been that of a phosphatase. This view has been fostered by results showing an elevation in DivK∼P in *pleC*::Tn*5* mutants [21]. Furthermore, *pleC_F778L_* mutants, which lack autokinase activity, appear to have a normal cell cycle [45]. However, later experiments have shown that, although cells possessing PleC_F778L_ progress through the cell cycle without any problems, they show a marked reduction in holdfast attachment [9]. These cells also show lower c-di-GMP levels, indicating that PleD is not sufficiently phosphorylated and activated in the absence of PleC kinase activity. Another mutant that reduces PleC autokinase activity is *divK_D90G_* [9]. In contrast to the *pleC_F778L_* mutants, cells possessing the *divK_D90G_* mutation do not require cytokinesis to initiate SPD.

**Figure 9:**
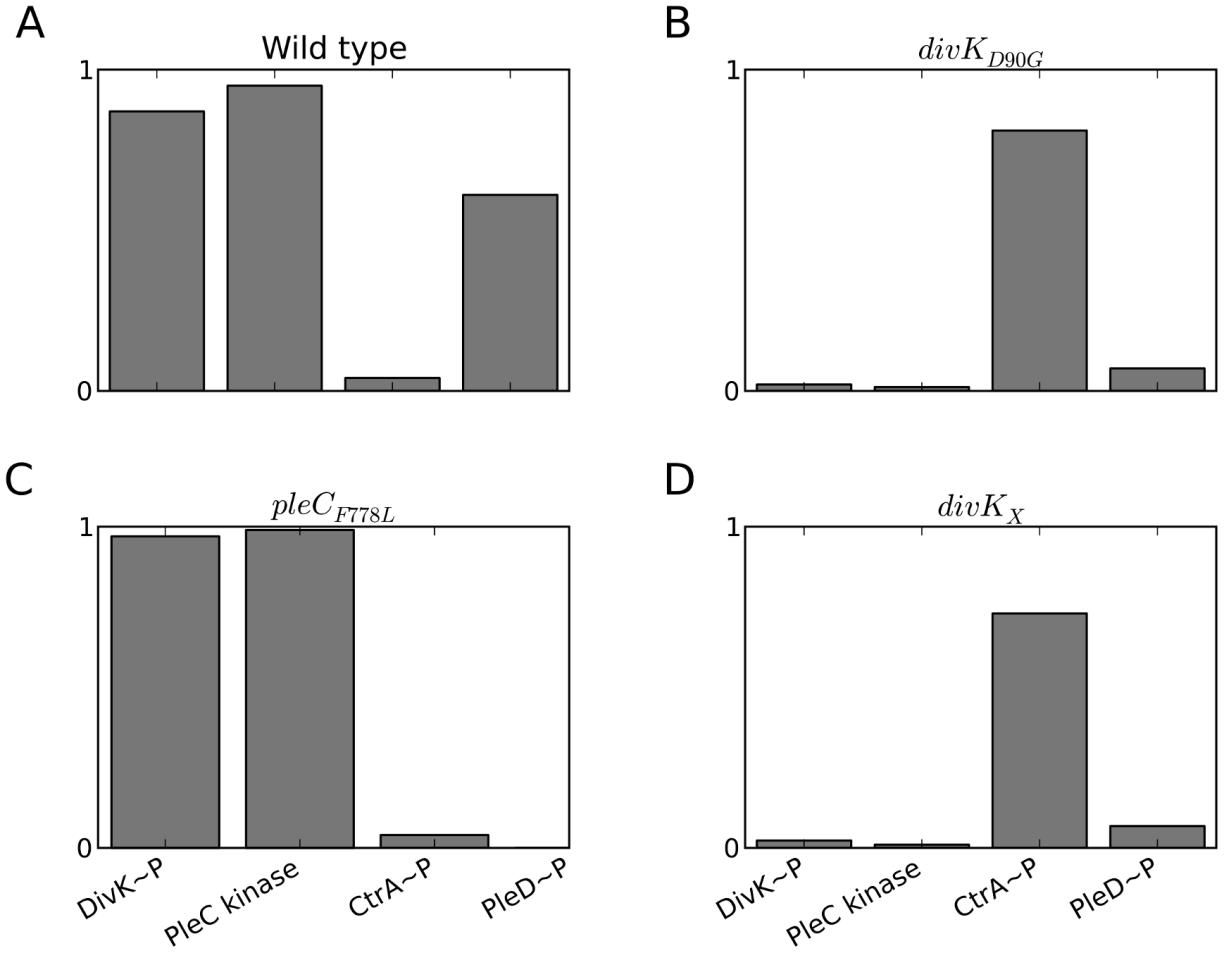
Comparison of *pleC*_F778L_ and *divK*_D90G_ mutants reveals the importance of the PleC kinase state. For wild-type cells (**A**) and three different mutants (**B–D**), we plot the steady-state fractions of four variables: PleC kinase, DivK∼P, CtrA∼P and PleD∼P. DivK_X_ is a hypothetical mutant form of DivK which is still phosphorylated and dephosphorylated by PleC but does not induce the conformational change of PleC from phosphatase to kinase.

If both mutations result in loss of PleC autokinase activity, why does only one of them exhibit premature SPD? One may argue that premature SPD is not due to the loss of PleC kinase activity, but is instead a consequence of inability of DivK_D90G_ to bind to DivL. However, we found that altering the rate constants governing the binding reaction had no effect on the phenotype, because DivK_D90G_∼P is low at all times and hence does not inhibit DivL. To shed light on this discrepancy, we propose a novel mutant strain of DivK, which we call *divK_X_*. The novel mutant deviates from *divK_D90G_* in that it retains wild type ability to bind to DivL. By simulations, we compare the phenotypes of *divK_D90G_*, *divK*_X_ and *pleC_F778L_* (see **Figure 9**). To model the *pleC_F778L_* mutant, we set the autophosphorylation rates to zero (**Table S8**).

In comparison to wild type, *pleC_F778L_* cells show a reduction in the level of PleD∼P; but DivK∼P and PleC kinase levels show only modest difference (**Figure 9A and C**). This simulated comparison agrees with experimental observations, which show that *pleC_F778L_* cells have reduced surface attachments but otherwise cycle normally. We reason that, although *pleC_F778L_* does not have kinase activity, it still retains its ability to switch to the kinase form. Hence, in stalked and pre-divisional cells, the majority of PleC is locked in the inactive kinase conformation. It follows that the PleC phosphatase to DivJ ratio is low and most of the DivK is phosphorylated. In comparison, *divK_D90G_* and a *divK_X_* show a reduction in the PleC kinase level **(** **Figure 9B and D** **)**. Since most PleC is in the phosphatase form, DivK∼P level is low and CtrA∼P level remains high throughout the cell cycle, thereby initiating SPD prematurely. Based on these simulation results, we propose that PleC kinase is important to prevent premature SPD. In the pre-divisional cell prior to compartmentalization, DivJ maintains PleC as a kinase while DivK is phosphorylated and bound to the pole/s. Once cytokinesis occurs, DivJ and PleC find themselves in different compartments, causing PleC to switch back to a phosphatase and allowing SPD.

## Discussion

We propose a model of the *Caulobacter* swarmer-to-stalked (G1-to-S) transition based on a pair of bifunctional histidine kinases, PleC and CckA. We suggest that the phosphatase-to-kinase transition of the PleC bifunctional enzyme is governed by concerted conformational changes brought about by homotropic interaction with its response regulator, DivK. By formulating a mathematical model based on a set of elementary chemical reactions, we show that the transition from phosphatase to kinase can function as a bistable switch driven by the starter kinase, DivJ. Our simulations reproduce the *in vitro* experimental observation that DivK and/or DivK∼P up-regulate PleC kinase activity. We hypothesize that even if DivK and DivK∼P have equal potential for causing the conformational change of PleC, DivK∼P is a more efficient inducer as a natural consequence of it being a substrate to the relaxed form, the phosphatase form of PleC. That DivK∼P is a more efficient inducer of the phosphatase-to-kinase transition creates a positive feedback loop and the potential for bistability, and bistability would explain why the swarmer-to-stalked transition is irreversible [35].

The swarmer-to-stalked transition is triggered by a rise in activity of the starter kinase DivJ. Evidence suggests that DivJ accumulates in response to nutritional signals [39]. Compared to well-fed cells, a greater fraction of *Caulobacter* cells are devoid of DivJ foci and exist as swarmer cells under conditions of glucose exhaustion. Hence, we consider DivJ as a nutritional proxy and use it as a control parameter in our model. As observed in our bifurcation diagrams, as total DivJ accumulates, the proteins that drive the swarmer-to-stalked transition show abrupt and irreversible changes in activity at the boundary of the bistable region. Once the transition has occurred, the control system will not permit a reverse transition (stalked-to-swarmer) in response to a marginal drop in nutritional level (i.e., in total DivJ concentration). In our view, once the PleC flips to the kinase form, the cell is committed to a new round of DNA synthesis before it can make a new motility apparatus in the pre-divisional stage. While bistability is not an essential feature of the morphological transitions in the *Caulobacter* division cycle, we propose that bistability in the PleC phosphatase-to-kinase transition may ensure that the swarmer-to-stalked transition is robust and does not undergo a reverse transition in response to small fluctuations in nutrient levels.

Our model is able to reproduce phenotypes of known experimental mutants and provide additional insight into the underlying physiology. Mutants overexpressing DivK show a decrease in CckA phosphorylation, in addition to filamentous growth and chromosomal over-replication [43]. Our two-parameter bifurcation diagrams indicate that cells with elevated DivK can no longer be regulated by DivJ. At higher concentrations, DivK can drive the positive feedback even in the absence of DivJ, resulting in PleC being in the kinase form and CtrA∼P being down-regulated. This prediction can be tested by overexpressing DivK in a Δ*divJ* background. Conversely Δ*divJ* mutants with a normal level of DivK are blocked in G1 phase owing to high CtrA∼P, while point mutations in *divL* and *cckA* rescue Δ*divJ* mutants [20]. Our simulations suggest that Δ*divJ* mutants can be rescued by point mutations that down-regulate CckA kinase activity, but not by mutants that up-regulate CckA's phosphatase activity.

Prior experiments and a mathematical model [46] dealing with the PleC-DivJ-DivK system have focused almost exclusively on the phosphatase form of PleC, while the kinase form has been considered inconsequential. We argue on the contrary that PleC kinase activity is important for proper progression through the *Caulobacter* cell cycle. To demonstrate this claim, we make an important distinction between two mutants *pleC_F778L_* and *divK_D90G_*. Our simulations show that while PleC_F778L_ has no autokinase activity, the majority of PleC_F778L_ molecules in stalked cells are in an inactive kinase form. These cells would therefore, appear normal. On the other hand, most PleC molecules remain in the phosphatase form in cells containing DivK_D90G_. We predict that in wild-type pre-divisional cells, PleC localized at the new pole is in the kinase form. Compartmentalization has the effect of withdrawing DivJ, causing PleC to switch back to the phosphatase form, as seen in our signal-response curves. The PleC-containing compartment, in the absence of DivJ, transitions into a swarmer cell. In mutant *divK_D90G_* cells, we predict that PleC at the new pole is always a phosphatase. This, we reason, would cause the premature presence of CtrA∼P in pre-divisional cells resulting in premature swarmer progeny-specific development (SPD). This conclusion is supported by the fact that filamentous *divK_D90G_* mutants show SPD in the absence of compartmentalization [45]. We are aware that *divK_D90G_* has a pleotropic effect of binding weakly to DivL. Hence, we hypothesize a novel mutant, *divK_X_*, which is similar to *divK_D90G_* but retains its ability to bind DivL. We simulate such a mutant and find its behavior to be comparable to *divK_D90G_*.

In this work, we are focusing on a small window in the *Caulobacter* cell cycle, the G1-to-S transition. We have not explored here how these coupled switches would function in a spatio-temporal context and whether they play a role in generating asymmetry in the two halves of the cell at a later stage in the division cycle. To explore these questions requires a spatio-temporal model that tracks the location of proteins in the cell and takes into account the effects of protein diffusion through the cytoplasm, as in [12], [46]. Without an accurate spatio-temporal model of these molecular interactions, we are still a long way from understanding the network of molecular interactions that governs the asymmetric life cycle of *Caulobacter crescentus*.

## Methods

The complete reaction network (**Figure S1**) was translated into a system of 52 non-linear ordinary differential equations (**Table S4**) using the mass-action law of chemical kinetics, with one exception. The mechanism by which DivL promotes the kinase form of CckA is unknown, so we modeled this step phenomenologically with a Hill function. Because there are many closed loops of elementary chemical reactions in **Figure S1**, we must choose rate constant values that respect the thermodynamic principle of detailed balance, as explained in **Text S1**. As long as we satisfy these thermodynamic constraints, we find that the reaction network exhibits bistability over a robust range of parameter values. The parameter values that we use for our simulations of the full model (**Table S4**) are given in **Table S5**.

The full model can be simplified slightly by reducing the first 28 equations in **Table S4** to the first 20 equations in **Table S6**, as explained in **Text S1**, section D, and confirmed in **Figure S4**.

The equations for both the full model and the reduced model were encoded as .ode files **(Text S2, S3, S4, S5)** and simulated using the freely available software, XPP-AUT. The signal-response curves were drawn using the AUTO facility of XPP-AUT. From the data points generated by XPP-AUT, the plots shown in the figures were generated using the python library, Matplotlib [47]. **Figure 3** is a simulation of the full model described in **Table S4**, while **Figures 4** **–** **9** are simulations of the reduced model and its corresponding mutants (**Table S4** and **Table S8**).

## Supporting Information

Figure S1 **Chemical reaction networks on which the model is based.** (**A**) PleC-DivK system. (**B**) DivJ-DivK and PleC-PleD system. (**C**) DivL-CckA-CtrA-CpdR system. (**D**) Synthesis and degradation of proteins. See **Table S1** for definitions of the protein complexes appearing in these figures.
(EPS)

Figure S2 **Schematic diagram of the free energy differences between the relaxed and tensed states of the free and ligand-bound enzyme.**
(EPS)

Figure S3 **(A) Baseline free energies assigned to intermediates in our model. (B) The common motif for all phospho-transfer reactions in our model.**
(EPS)

Figure S4 **Wiring diagram illustrating the elementary chemical reactions in the reduced model.**
(EPS)

Figure S5 **Bistability properties of the reduced version of the DivJ-PleC-DivK model are similar to the full-sized model.** The one-parameter bifurcation diagrams compare the steady state values of (**A**) PleC kinase, (**B**) DivK∼P, and (**C**) PleD∼P between the full-sized (blue line) and reduced (green line) versions of the model.
(TIFF)

Figure S6 **The swarmer-to-stalked transition is accompanied by modest changes to the total concentrations of regulatory proteins but significant changes to their phosphorylation states.** The one-parameter bifurcation diagrams on the left show the total concentration of (**A**) DivK, (**C**) CtrA, and (**E**) PleD as functions of total DivJ. On the right, the one-parameter bifurcation diagrams show the phosphorylated fraction of the total concentration for (**B**) DivK, (**D**) CtrA, and (**F**) PleD. Red line, fraction that is phosphorylated and free; green line, phosphorylated fraction both free and bound.
(PDF)

Table S1 **Description of species in the model with their abbreviated names.**
(DOCX)

Table S2 **Representative reactions in the model.**
(DOCX)

Table S3 **Gibbs free energy change for DivK binding to PleC.**
(DOCX)

Table S4 **Equations governing the full model.**
(DOCX)

Table S5 **Basal parameter values used in the full model.**
(DOCX)

Table S6 **Equations governing the reduced model.**
(DOCX)

Table S7 **Basal parameter values used in the reduced model.**
(DOCX)

Table S8 **Parameter values used to simulate mutants.**
(DOCX)

Text S1 **Detailed description of model formulation.**
(DOCX)

Text S2 **An ODE file of the full-size model to simulate the wild-type results.**
(TXT)

Text S3 **An ODE file of the reduced model to simulate the wild-type results.**
(TXT)

Text S4 **An ODE file of the reduced model to simulate the** ***divK_D90G_*** **mutant.**
(TXT)

Text S5 **An ODE file of the reduced model to simulate the** ***pleC_F778L_*** **mutant.**
(TXT)
