# Supplementary figures and images for: Potential Role of a Bistable Histidine Kinase Switch in the Asymmetric Division Cycle of Caulobacter crescentus
Source: PLoS Comput Biol. 2013 Sep 12;9(9):e1003221. doi: 10.1371/journal.pcbi.1003221 (PMC3772055; doi:10.1371/journal.pcbi.1003221)

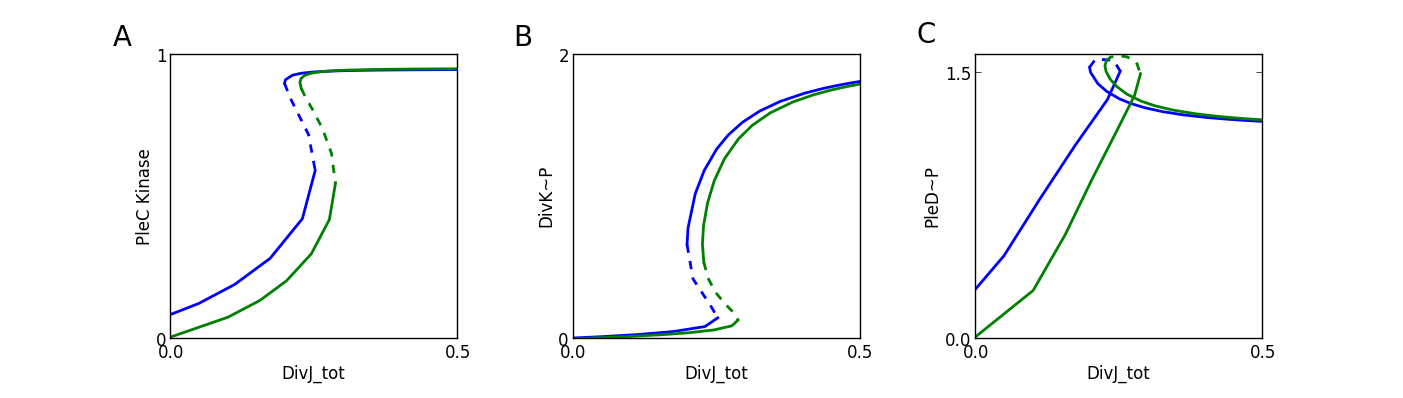

Supplement: Figure S5 — Bistability properties of the reduced version of the DivJ-PleC-DivK model are similar to the full-sized model. The one-parameter bifurcation diagrams compare the steady state values of (A) PleC kinase, (B) DivK∼P, and (C) PleD∼P between the full-sized (blue line) and reduced (green line) versions of the model. (TIFF) [file pcbi.1003221.s005.tiff]

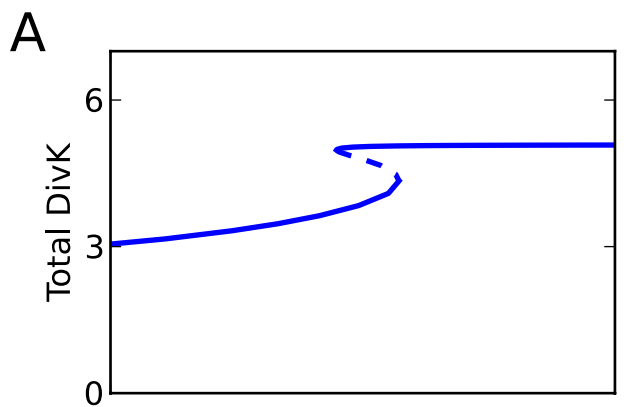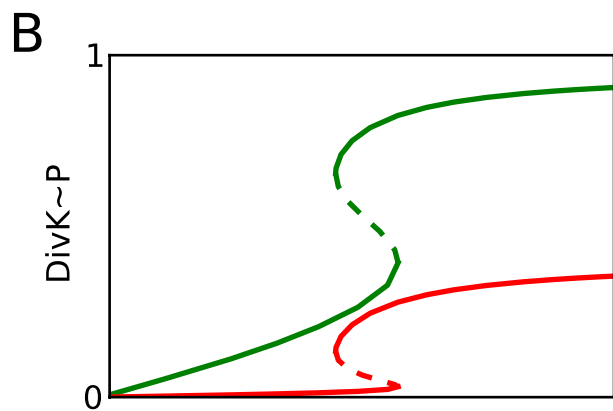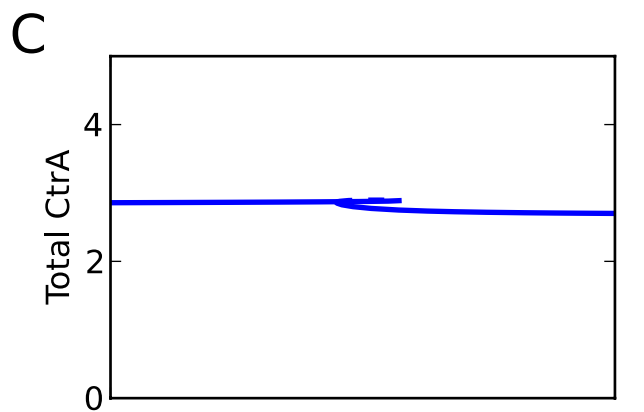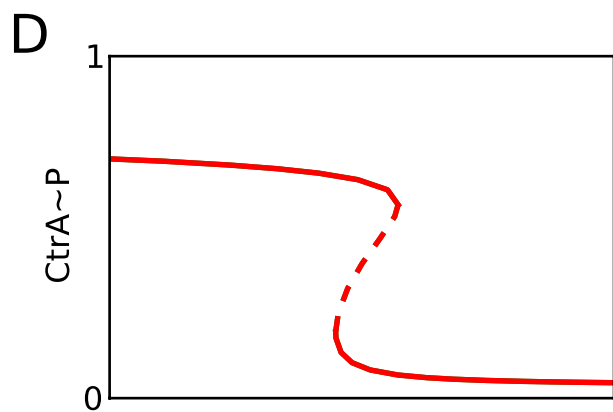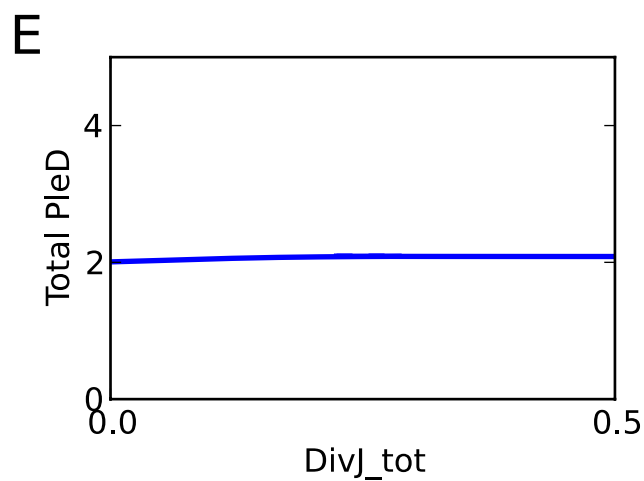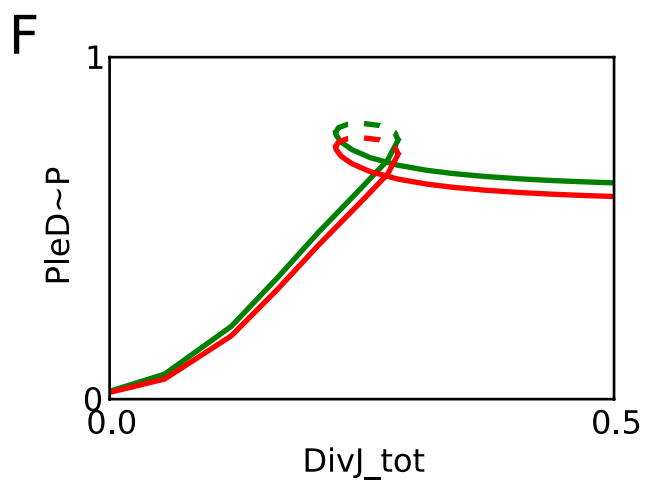

Supplement: Figure S6 — The swarmer-to-stalked transition is accompanied by modest changes to the total concentrations of regulatory proteins but significant changes to their phosphorylation states. The one-parameter bifurcation diagrams on the left show the total concentration of (A) DivK, (C) CtrA, and (E) PleD as functions of total DivJ. On the right, the one-parameter bifurcation diagrams show the phosphorylated fraction of the total concentration for (B) DivK, (D) CtrA, and (F) PleD. Red line, fraction that is phosphorylated and free; green line, phosphorylated fraction both free and bound. (PDF) [file pcbi.1003221.s006.pdf]
