# Supplementary material for: Potential Role of a Bistable Histidine Kinase Switch in the Asymmetric Division Cycle of Caulobacter crescentus
Source: PLoS Comput Biol. 2013 Sep 12;9(9):e1003221. doi: 10.1371/journal.pcbi.1003221 (PMC3772055; doi:10.1371/journal.pcbi.1003221)
Supplement: Table S1 — Description of species in the model with their abbreviated names. (DOCX) [file pcbi.1003221.s007.docx]

| **Table S1**: Description of species in the model with their abbreviated names | | |
| --- | --- | --- |
| DivK~P |  | dk |
| DivK |  | dkp |
| PleC | PleC:PleC (phosphatase) | pc |
| PleC_ph1_ | PleC:PleC:DivK~P | ph1 |
| PleC_ph2_ | PleC:PleC:DivK | ph2 |
| PleC_ph11_ | DivK~P:PleC:PleC:DivK~P (phosphatase) | ph11 |
| PleC_ph12_ | DivK:PleC:PleC:DivK~P (phosphatase) | ph12 |
| PleC_ph22_ | DivK:PleC:PleC:DivK (phosphatase) | ph22 |
| PleC_ph1p_ | PleC:PleC~P (phosphatase form) | ph1p |
| PleC_ph2p_ | PleC~P:PleC~P (phosphatase form) | ph2p |
| PleC_kin_ | PleC:PleC (kinase) | pck |
| PleC_pkdk_ | PleC:PleC:DivK | pkdk |
| PleC_kin11_ | DivK~P: PleC:PleC: DivK~P (kinase) | pk11 |
| PleC_kin12_ | DivK: PleC:PleC: DivK~P (kinase) | pk12 |
| PleC_kin22_ | DivK:PleC: PleC: DivK (kinase) | pk22 |
| PleC_kin0_ | DivK~P: PleC~P:PleC~P: DivK~P | pk0 |
| PleC_kin1_ | PleC~P:PleC~P: DivK~P | pk1 |
| PleC_kin2_ | DivK: PleC~P:PleC~P: DivK~P | pk2 |
| PleC_kin3_ | PleC~P:PleC~P: DivK | pk3 |
| PleC_kin4_ | DivK: PleC~P:PleC~P: DivK | pk4 |
| PleC_pt2_ | DivK~P: PleC:PleC~P: DivK~P | pt2 |
| PleC_pt3_ | PleC~P:PleC: DivK~P | pt3 |
| PleC_pt4_ | DivK:PleC~P:PleC: DivK~P | pt4 |
| PleC_pt3h_ | DivK~P:PleC:PleC | pt3h |
| PleC_k1h_ | PleC:PleC~P: DivK~P | pk1h |
| PleC_k3h_ | DivK:PleC~P:PleC | pk3h |
| PleC_kin1p_ | PleC:PleC~P (kinase form) | pk1p |
| PleC_kin2p_ | PleC~P:PleC~P (kinase form) | pk2p |
| PleD |  | pld |
| PleD~P |  | pldp |
| PleC_kin5_ | DivK~P:PleC~P:PleC~P:PleD | pk5 |
| PleC_kin6_ | DivK:PleC~P:PleC~P:PleD | pk6 |
| PleC_pt5_ | DivK~P:PleC~P:PleC:PleD~P | pt5 |
| PleC_pt6_ | DivK:PleC~P:PleC:PleD~P | pt6 |
| DivJ |  | dj |
| DivK:DivK |  | jk |
| DivL |  | dl |
| DivL:DivK~P | DivL:DivK~P | dldk |
| CtrA |  | ctr |
| CtrA~P |  | ctrp |
| CpdR |  | cpd |
| CpdR~P |  | cpdp |
| CckA _kin_ |  | ck |
| CckA _phos_ |  | cp |
| CckA _kin1_ | CckA~P:CtrA | ck1 |
| CckA _kin2_ | CckA~P:CpdR | ck2 |
| CckA _ct1_ | CckA:CtrA~P (kinase) | ct1 |
| CckA _ct2_ | CckA:CpdR~P (kinase) | ct2 |
| CckA _ph1_ | CckA:CtrA~P (phosphatase) | ch1 |
| CckA _ph2_ | CckA:CpdR~P (phosphatase) | ch2 |
| Phos | Generic phosphatase | phos |
