# Supplementary material for: Potential Role of a Bistable Histidine Kinase Switch in the Asymmetric Division Cycle of Caulobacter crescentus
Source: PLoS Comput Biol. 2013 Sep 12;9(9):e1003221. doi: 10.1371/journal.pcbi.1003221 (PMC3772055; doi:10.1371/journal.pcbi.1003221)
Supplement: Table S2 — Representative reactions in the model. (DOCX) [file pcbi.1003221.s008.docx]

| **Table S2.** Representative reactions in the model | | | |
| --- | --- | --- | --- |
| **Phosphorylation reactions** |  |  |  |
| $ADP +P_{\text{i}}\to ATP +H_{\text{2}}\text{O}$ | $\Delta G_{\text{ATP}}^{0}= +50 kJ/mole$ |  | (R1) |
| $\mathrm{PleC}_{\text{kin}} +P_{\text{i}}\to\mathrm{PleC}_{\text{kin}}\text{\textasciitilde P} +H_{\text{2}}\text{O}$ | $\Delta G_{\text{PleC\textasciitilde P}}^{0}= +50 kJ/mole$ |  | (R2) |
| $DivK +P_{\text{i}}\to DivK\sim P +H_{\text{2}}\text{O}$ | $\Delta G_{\text{DivK\textasciitilde P}}^{0}= +30 kJ/mole$ |  | (R3) |
| **R_0_ 🡪 T_0_ reaction** |  |  |  |
| $\mathrm{PleC}_{\text{pho}} \to\mathrm{PleC}_{\text{kin}}$ | $\Delta G_{\text{RT}}^{0}= +8.55 kJ/mole$ | $K_{\mathrm{eq}}=0.0316$ | (R4) |
| **Typical ligand binding reactions** |  |  |  |
| $\mathrm{PleC}_{\text{pho}}+DivK\sim P \to\mathrm{PleC}_{\mathrm{pho}}: DivK\sim P$ | $\Delta G_{\mathrm{HS}}^{0}+\Delta G_{\mathrm{HL}}^{0}= 0 kJ/mole$ | $K_{\mathrm{eq}}=1$ | (R5) |
| $\mathrm{PleC}_{\text{pho}} +DivK \to\mathrm{PleC}_{\mathrm{pho}}: DivK$ | $\Delta G_{\mathrm{HP}}^{0}+\Delta G_{\mathrm{HL}}^{0}= +11.4 kJ/mole$ | $K_{\mathrm{eq}}=0.01$ | (R6) |
| $\mathrm{PleC}_{\text{kin}} +DivK\sim P \to\mathrm{PleC}_{\mathrm{kin}}: DivK\sim P$ | $\Delta G_{\mathrm{KP}}^{0}+\Delta G_{\mathrm{KL}}^{0}= -8.55 kJ/mole$ | $K_{\mathrm{eq}}=31.6$ | (R7) |
| $\mathrm{PleC}_{\text{kin}} +DivK \to\mathrm{PleC}_{\mathrm{kin}}: DivK$ | $\Delta G_{\mathrm{KS}}^{0}+\Delta G_{\mathrm{KL}}^{0}= -19.95 kJ/mole$ | $K_{\mathrm{eq}}=3160$ | (R8) |
| **Phosphatase reaction** |  |  |  |
| $\mathrm{PleC}_{\mathrm{pho}}:DivK\sim P +H_{2}O\to\mathrm{PleC}_{\mathrm{pho}}:DivK+P_{\text{i}}$ | $\Delta G_{\text{ph}}^{0}= -18.6 kJ/mole$ | $K_{\mathrm{eq}}=1830$ | (R9) |
| **Phosphotransfer reaction** |  |  |  |
| $\mathrm{PleC}_{\text{kin}}\sim P:DivK \to\mathrm{PleC}_{\mathrm{kin}}:DivK\sim P$ | $\Delta G_{\text{pt}}^{0}= -8.6 kJ/mole$ | $K_{\mathrm{eq}}=32$ | (R10) |
| **Auto-dephosphorylation reaction** |  |  |  |
| $\mathrm{PleC}_{\text{pho}}\sim P +H_{2}O \to\mathrm{PleC}_{\mathrm{pho}}+P_{\text{i}}$ | $\Delta G_{\text{ad}}^{0}= -50 kJ/mole$ | irreversible | (R11) |
