# Supplementary material for: Potential Role of a Bistable Histidine Kinase Switch in the Asymmetric Division Cycle of Caulobacter crescentus
Source: PLoS Comput Biol. 2013 Sep 12;9(9):e1003221. doi: 10.1371/journal.pcbi.1003221 (PMC3772055; doi:10.1371/journal.pcbi.1003221)
Supplement: Table S3 — Gibbs free energy change for DivK binding to PleC. (DOCX) [file pcbi.1003221.s009.docx]

| **Table S3.** Gibbs free energy changes for DivK binding to PleC | | |
| --- | --- | --- |
| $Formation of phosphatase\text{-}substrate complex$ | $\Delta G_{\mathrm{HS}}^{0}= 0 kJ/mole$ | $K_{\text{eq}}= 1$ |
| $Formation of kinase\text{-}substrate complex$ | $\Delta G_{\mathrm{KS}}^{0}= -5.7 kJ/mole$ | $K_{\text{eq}}= 10$ |
| $Formation of phosphatase\text{-}product complex$ | $\Delta G_{\mathrm{HP}}^{0}= +11.4 kJ/mole$ | $K_{\text{eq}}= 0.01$ |
| $Formation of kinase\text{-}product complex$ | $\Delta G_{\mathrm{KP}}^{0}= +5.7 kJ/mole$ | $K_{\text{eq}}= 0.1$ |
| $Ligand binding to phosphatase (relaxed form)$ | $\Delta G_{\mathrm{HL}}^{0}= 0 kJ/mole$ | $K_{\text{eq}}= 1$ |
| $Ligand binding to kinase (tensed form)$ | $\Delta G_{\mathrm{KL}}^{0}= -14.25 kJ/mole$ | $K_{\text{eq}}= 316$ |
