# Supplementary material for: Potential Role of a Bistable Histidine Kinase Switch in the Asymmetric Division Cycle of Caulobacter crescentus
Source: PLoS Comput Biol. 2013 Sep 12;9(9):e1003221. doi: 10.1371/journal.pcbi.1003221 (PMC3772055; doi:10.1371/journal.pcbi.1003221)
Supplement: Table S5 — Basal parameter values used in the full model. (DOCX) [file pcbi.1003221.s011.docx]

| **Table S5:** Basal parameter values used in the full model* | | | | | | | |
| --- | --- | --- | --- | --- | --- | --- | --- |
| *k*_ph1-ph11_ | 5 | *k*_ph11-ph1_ | 5 | *k*_ph11-pk11_ | 5 | *k*_pk11-ph11_ | 5 |
| *k*_pk11-pk0_ | 5 | *k*_pk0-pk11_ | 5 | *k*_pk0-pk1_ | 0.16 | *k*_pk1-pk0_ | 5 |
| *k*_pk1-pk2_ | 5 | *k*_pk2-pk1_ | 0.0016 | *k*_pk1-pk1h_ | 5 | *k*_pk1h-pk1_ | 5 |
| *k*_pk1-pk2p_ | 0.16 | *k*_pk2p-pk1_ | 5 | *k*_pk2-pt2_ | 5 | *k*_pt2-pk2_ | 0.16 |
| *k*_pt2-pk1h_ | 0.16 | *k*_pk1h-pt2_ | 5 | *k*_pk2p-ph2p_ | 5 | *k*_ph2p-pk2p_ | 0.005 |
| *k*_ph2p-pc_ | 5 | *k*_ph1p-pc_ | 5 | *k*_pk3-pk2p_ | 0.0016 | *k*_pk2p-pk3_ | 5 |
| *k*_pk2-pk3_ | 0.16 | *k*_pk3-pk2_ | 5 | *k*_pk3-pt3_ | 5 | *k*_pt3-pk3_ | 0.16 |
| *k*_pt3-pk1p_ | 0.16 | *k*_pk1p-pt3_ | 5 | *k*_pk1p-pk1h_ | 5 | *k*_pk1h-pk1p_ | 0.16 |
| *k*_pk3-pk4_ | 5 | *k*_pk4-pk3_ | 0.0016 | *k*_pk4-pt4_ | 5 | *k*_pt4-pk4_ | 0.16 |
| *k*_pt4-pk11_ | 5 | *k*_pk11-pt4_ | 0.16 | *k*_pc-ph2_ | 0.05 | *k*_ph2-pc_ | 5 |
| *k*_ph2-ph22_ | 0.05 | *k*_ph22-ph2_ | 5 | *k*_ph22-pk22_ | 5 | *k*_pk22-ph22_ | 5e-08 |
| *k*_pk22-pk4_ | 5 | *k*_pk4-pk22_ | 5 | *k*_ph2-ph12_ | 5 | *k*_ph12-ph2_ | 5 |
| *k*_ph1-ph12_ | 0.05 | *k*_ph12-ph1_ | 5 | *k*_ph12-pk12_ | 5 | *k*_pk12-ph12_ | 5e-04 |
| *k*_pk12-pk2_ | 5 | *k*_pk2-pk12_ | 5 | *k*_pch-pck_ | 0.005 | *k*_pck-pch_ | 5 |
| *k*_pck-pkdp_ | 5 | *k*_pkdk-pck_ | 0.0016 | *k*_pkdk-pk22_ | 5 | *k*_pk22-pkdk_ | 0.0016 |
| *k*_pkdk-pk12_ | 5 | *k*_pk12-pkdk_ | 0.16 | *k*_pck-pt3h_ | 5 | *k*_pt3h-pck_ | 0.16 |
| *k*_pt3h-pk11_ | 5 | *k*_pk11-pt3h_ | 0.16 | *k*_pt3h-pk12_ | 5 | *k*_pk12-pt3h_ | 0.0016 |
| *k*_pt4-pk3h_ | 0.16 | *k*_pk3h-pt4_ | 5 | *k*_pk3-pk3h_ | 5 | *k*_pk3h-pk3_ | 5 |
| *k*_pk1p-pk3h_ | 5 | *k*_pk3h-pk1p_ | 0.0016 | *k*_pk3h-pt3h_ | 5 | *k*_pt3h-pk3h_ | 0.16 |
| *k*_pk1p-ph1p_ | 5 | *k*_ph1p-pk1p_ | 0.005 | *k*_syn-dk_ | 0.015 | *k*_deg-dk_ | 0.005 |
| *k*_deg-dkp_ | 0.005 | *k*_j-jk_ | 1 | *k*_jk-j_ | 0.1 | *k*_ph1-ph2_ | 10 |
| *k*_ph2-ph1_ | 5e-03 | *k*_pc-ph1_ | 5 | *k*_ph1-pc_ | 5 | *k*_syn-pld_ | 0.01 |
| *k*_deg-pld_ | 0.005 | *k*_deg-pldp_ | 0.005 | *k*_phos-ph3_ | 1 | *k*_ph3-phos_ | 0.1 |
| *k*_pk1-pk5_ | 10 | *k*_pk5-pk1_ | 0.31 | *k*_pk5-pt5_ | 10 | *k*_pt5-pk5_ | 0.31 |
| *k*_pt5-pk1h_ | 10 | *k*_pk1h-pt5_ | 0.31 | *k*_pk3-pk6_ | 10 | *k*_pk6-pk3_ | 0.31 |
| *k*_pk6-pt6_ | 10 | *k*_pt6-pk6_ | 0.31 | *k*_pt6-pk3h_ | 10 | *k*_pk3h-pt6_ | 0.31 |
| *k*_e-ph3_ | 0.2 |  |  |  |  |  |  |
| *k*_dl-dldk_ | 1 | *k*_dldk-dl_ | 0.1 | *k*_syndl_ | 0.005 | *k*_degdl_ | 0.005 |
| *k*_cp-ck_ | 1 | *k*_cp-ck_ | 0.1 | *k*_cp-ch2_ | 1 | *k*_ch2-cp_ | 0.1 |
| *k*_syn-ctra_ | 0.05 | *k*_deg-ctr_ | 0.02 | *k*_deg-ctrp_ | 0.02 | *k*_syn-cpdr_ | 0.05 |
| *k*_deg-cpdr_ | 0.02 | *k*_deg-cpdp_ | 0.02 | *k*_ck-ck1_ | 1 | *k*_ck1-ck_ | 0.1 |
| *k*_ck1-ct1_ | 1 | *k*_ct1-ck1_ | 0.1 | *k*_ct1-ck_ | 1 | *k*_ck-ct1_ | 0.1 |
| *k*_cp-ch1_ | 1 | *k*_ch1-cp_ | 0.1 | *k*_e-ch1_ | 1 | *k*_ck-ck2_ | 1 |
| *k*_ck2-ck_ | 0.1 | *k*_ck2-ct2_ | 1 | *k*_ct2-ck2_ | 0.1 | *k*_ct2-ck_ | 1 |
| *k*_ck-ct2_ | 0.1 | *k*_e-ch2_ | 1 |  |  |  |  |
| DivJ_tot | 0.5 | PleC_tot | 1 | CckA_tot | 1 | Phos_tot | 0.1 |
| *K*_dl_ | 0.75 |  |  |  |  |  |  |
| *For each reaction of the form $X\to Y,$ the parameters *k*_x-y_ and *k*_y-x_ are the forward and reverse rate constants (min^-1^). DivJ_tot, PleC_tot, CckA_tot, Phos_tot and *K*_dl_ are dimensionless constants representing concentrations. | | | | | | | |
