# Supplementary material for: Potential Role of a Bistable Histidine Kinase Switch in the Asymmetric Division Cycle of Caulobacter crescentus
Source: PLoS Comput Biol. 2013 Sep 12;9(9):e1003221. doi: 10.1371/journal.pcbi.1003221 (PMC3772055; doi:10.1371/journal.pcbi.1003221)
Supplement: Table S6 — Equations governing the reduced model. (DOCX) [file pcbi.1003221.s012.docx]

| **Table S6 :** Equations governing the reduced model | |
| --- | --- |
| $\frac{d[DivK\sim P]}{dt} = -k_{deg-dkp}\cdot\left[ DivK\sim P \right]- k_{pc-ph1}\cdot\left[ \mathrm{PleC} \right]\cdot\left[ DivK\sim P \right]+k_{ph1-pc}\cdot\left[ \mathrm{Ple}C_{h1} \right]-k_{ph1-pk11}\cdot\left[ \mathrm{Ple}C_{ph1} \right]\cdot\left[ DivK\sim P \right]+k_{pk11-ph1}\cdot\left[ \mathrm{PleC}_{kin11} \right] -k_{ph2-pk12}\cdot\left[ \mathrm{Ple}C_{ph2} \right]\cdot\left[ DivK\sim P \right]+ k_{pk12-ph2}\cdot\left[ \mathrm{Ple}C_{kin12} \right]-k_{pk1-pk0}\cdot\left[ \mathrm{Ple}C_{kin1} \right]\cdot\left[ DivK\sim P \right]+k_{pk0-pk1}\cdot\left[ \mathrm{Ple}C_{kin0} \right]- k_{pk3-pk2}\cdot\left[ \mathrm{Ple}C_{kin3} \right]\cdot\left[ DivK\sim P \right]+ k_{pk2-pk3}\cdot\left[ \mathrm{Ple}C_{kin2} \right] + k_{pt2-pk1h}\cdot\left[ \mathrm{Ple}C_{pt2} \right]-k_{pk1h-pt2}\cdot\left[ \mathrm{Ple}C_{kin1h} \right]\cdot\left[ DivK\sim P \right]-k_{pk2p-pk1} \cdot\left[ \mathrm{Ple}C_{kin2p} \right]\cdot\left[ DivK\sim P \right]+ k_{pk1-pk2p}\cdot\left[ \mathrm{Ple}C_{kin1} \right]- k_{pk1p-pk1h}\cdot\left[ \mathrm{Ple}C_{kin1p} \right]\cdot\left[ DivK\sim P \right]+ k_{pk1h-pk1p}\cdot\left[ \mathrm{Ple}C_{kin1h} \right]+k_{pt3-pk1p} \cdot\left[ \mathrm{Ple}C_{pt3} \right]- k_{pk1p-pt3}\cdot\left[ \mathrm{Ple}C_{kin1p} \right]\cdot\left[ DivK\sim P \right]+ k_{e-jk}\cdot\left[ DivJ:DivK \right] - k_{pk3h-pt4}\cdot\left[ \mathrm{PleC}_{kin3h} \right]\cdot\left[ DivK\sim P \right]+ k_{pt4-pk3h}\cdot\left[ \mathrm{PleC}_{pt4} \right]+ k_{h1-h2}\cdot\left[ \mathrm{Ple}C_{ph1} \right]\cdot\left[ \mathrm{DivK} \right]- k_{h2-h1}\cdot\left[ \mathrm{Ple}C_{ph2} \right]\cdot\left[ DivK\sim P \right]-k_{dl-dldk}\cdot\left[ \mathrm{DivL} \right]\cdot\left[ DivK\sim P \right]+ k_{dldk-dl}\cdot\left[ DivL:DivK\sim P \right]$ | 1 |
| $\frac{d\left[ \mathrm{DivK} \right]}{dt} = k_{syn-dk}-k_{deg-dk}\cdot\left[ \mathrm{DivK} \right]- k_{pc-ph2}\cdot\left[ \mathrm{PleC} \right]\cdot\left[ \mathrm{DivK} \right]+k_{ph2-pc}\cdot\left[ \mathrm{Ple}C_{h2} \right]- k_{ph2-pk22}\cdot\left[ \mathrm{Ple}C_{ph2} \right]\cdot\left[ \mathrm{DivK} \right]+ k_{pk22-ph2}\cdot\left[ \mathrm{PleC}_{kin22} \right]-k_{ph1-pk12}\cdot\left[ \mathrm{Ple}C_{ph1} \right]\cdot\left[ \mathrm{DivK} \right]+ k_{pk12-ph1}\cdot\left[ \mathrm{Ple}C_{kin12} \right]-k_{pk1-pk2}\cdot\left[ \mathrm{Ple}C_{kin1} \right]\cdot\left[ \mathrm{DivK} \right]+k_{pk2-pk1}\cdot\left[ \mathrm{Ple}C_{kin2} \right]- k_{pk3-pk4}\cdot\left[ \mathrm{Ple}C_{kin3} \right]\cdot\left[ \mathrm{DivK} \right]+ k_{pk4-pk3}\cdot\left[ \mathrm{Ple}C_{kin4} \right]- k_{pk2p-pk3}\cdot\left[ \mathrm{Ple}C_{pk2p} \right]\cdot\left[ \mathrm{DivK} \right]+k_{pk3-pk2p}\cdot\left[ \mathrm{Ple}C_{kin3} \right]-k_{j-jk}\cdot\left[ \mathrm{DivJ} \right]\cdot\left[ \mathrm{DivK} \right]+ k_{jk-j}\cdot\left[ DivJ:DivK \right]- k_{ph1-pk12}\cdot\left[ \mathrm{Ple}C_{h1} \right]\cdot\left[ \mathrm{DivK} \right]+ k_{pk12-ph1}\cdot\left[ \mathrm{Ple}C_{\mathrm{kin}12} \right]- k_{pk1p-pk3h}\cdot\left[ \mathrm{Ple}C_{kin1p} \right]\cdot\left[ \mathrm{DivK} \right]+k_{pk3h-pk1p}\cdot\left[ \mathrm{Ple}C_{kin3h} \right]- k_{h1-h2}\cdot\left[ \mathrm{Ple}C_{ph1} \right]\cdot\left[ \mathrm{DivK} \right]+ k_{h2-h1}\cdot\left[ \mathrm{Ple}C_{ph2} \right]\cdot\left[ DivK\sim P \right]$ | 2 |
| $\frac{d\left[ \mathrm{PleC} \right]}{dt} = - k_{pc-ph2}\cdot\left[ \mathrm{PleC} \right]\cdot\left[ \mathrm{DivK} \right]+k_{ph2-pc}\cdot\left[ \mathrm{Ple}C_{h2} \right] - k_{pc-ph1}\cdot\left[ \mathrm{PleC} \right]\cdot\left[ DivK\sim P \right]+k_{ph1-pc}\cdot\left[ \mathrm{Ple}C_{h1} \right]+k_{ph2p-pc}\cdot\left[ \mathrm{Ple}C_{h2p} \right]+k_{ph1p-pc}\cdot\left[ \mathrm{Ple}C_{h1p} \right]$ | 3 |
| $\frac{d\left[ \mathrm{PleC}_{ph1} \right]}{dt} = k_{pc-ph1}\cdot\left[ \mathrm{PleC} \right]\cdot\left[ DivK\sim P \right]-k_{ph1-pc}\cdot\left[ \mathrm{Ple}C_{h1} \right]- k_{ph1-pk11}\cdot\left[ \mathrm{Ple}C_{ph1} \right]\cdot\left[ DivK\sim P \right]+ k_{pk11-ph1}\cdot\left[ \mathrm{PleC}_{kin11} \right] - k_{ph1-pk12}\cdot\left[ \mathrm{Ple}C_{ph1} \right]\cdot\left[ \mathrm{DivK} \right] + k_{pk12-ph1}\cdot\left[ \mathrm{Ple}C_{kin12} \right]-k_{h1-h2}\cdot\left[ \mathrm{Ple}C_{ph1} \right]\cdot\left[ \mathrm{DivK} \right]+k_{h2-h1}\cdot\left[ \mathrm{Ple}C_{ph2} \right]\cdot\left[ DivK\sim P \right]-k_{ph1-ph2}\cdot\left[ \mathrm{Ple}C_{ph1} \right]+k_{ph2-ph1}\cdot\left[ \mathrm{Ple}C_{ph2} \right]$ | 4 |
| $\frac{d\left[ \mathrm{PleC}_{ph2} \right]}{dt} = k_{pc-ph2}\cdot\left[ \mathrm{PleC} \right]\cdot\left[ \mathrm{DivK} \right]-k_{ph2-pc}\cdot\left[ \mathrm{Ple}C_{ph2} \right] - k_{ph2-pk22}\cdot\left[ \mathrm{Ple}C_{ph2} \right]\cdot\left[ \mathrm{DivK} \right]+ k_{pk22-ph2}\cdot\left[ \mathrm{PleC}_{kin22} \right] -k_{ph2-pk12}\cdot\left[ \mathrm{Ple}C_{ph2} \right]\cdot\left[ DivK\sim P \right]+ k_{pk12-ph2}\cdot\left[ \mathrm{Ple}C_{kin12} \right]+k_{h1-h2}\cdot\left[ \mathrm{Ple}C_{ph1} \right]\cdot\left[ \mathrm{DivK} \right]-k_{h2-h1}\cdot\left[ \mathrm{Ple}C_{ph2} \right]\cdot\left[ DivK\sim P \right] +k_{ph1-ph2}\cdot\left[ \mathrm{Ple}C_{ph1} \right]-k_{ph2-ph1}\cdot\left[ \mathrm{Ple}C_{ph2} \right]$ | 5 |
| ${\frac{d\left[ \mathrm{PleC}_{kin11} \right]}{dt} =k_{ph1-p11}\cdot\left[ \mathrm{Ple}C_{ph1} \right]\cdot\left[ DivK\sim P \right]-k_{pk11-ph1}\cdot\left[ \mathrm{PleC}_{kin11} \right]- k_{pk11-pk0}\cdot\left[ \mathrm{PleC}_{kin11} \right]+ k_{pk0-pk11}\cdot\left[ \mathrm{PleC}_{kin0} \right]+ k}_{pt4-pk11}\cdot\left[ \mathrm{PleC}_{pt4} \right]- k_{pk11-pt4}\cdot\left[ \mathrm{PleC}_{kin11} \right]$ | 6 |
| $\frac{d\left[ \mathrm{PleC}_{kin12} \right]}{dt} =k_{ph2-pk12}\cdot\left[ \mathrm{Ple}C_{ph2} \right]\cdot\left[ DivK\sim P \right]- k_{pk12-ph2}\cdot\left[ \mathrm{Ple}C_{kin12} \right]+ k_{ph1-pk12}\cdot\left[ \mathrm{Ple}C_{ph1} \right]\cdot\left[ \mathrm{DivK} \right]- k_{pk12-ph1}\cdot\left[ \mathrm{Ple}C_{kin12} \right]- k_{pk22-pk2}\cdot\left[ \mathrm{PleC}_{kin22} \right]+ k_{pk2-pk22}\cdot\left[ \mathrm{PleC}_{kin2} \right]$ | 7 |
| ${\frac{d\left[ \mathrm{PleC}_{kin22} \right]}{dt} =k_{ph2-pk22}\cdot\left[ \mathrm{Ple}C_{h2} \right]\cdot\left[ \mathrm{DivK} \right]- k_{pk22-ph2}\cdot\left[ \mathrm{PleC}_{kin22} \right]}- k_{p22-pk4}\cdot\left[ \mathrm{PleC}_{kin22} \right]+ k_{pk4-pk22}\cdot\left[ \mathrm{PleC}_{kin4} \right]$ | 8 |
| $\frac{d\left[ \mathrm{PleC}_{kin0} \right]}{dt} = k_{pk11-pk0}\cdot\left[ \mathrm{PleC}_{kin11} \right]- k_{pk0-pk11}\cdot\left[ \mathrm{PleC}_{kin0} \right] - k_{pk0-pk1}\cdot\left[ \mathrm{PleC}_{kin0} \right]+ k_{pk1-pk0}\cdot\left[ \mathrm{PleC}_{kin1} \right]\cdot\left[ DivK\sim P \right]$ | 9 |
| $\frac{d\left[ \mathrm{PleC}_{kin2} \right]}{dt} = k_{pk12-pk2}\cdot\left[ \mathrm{PleC}_{kin12} \right]-k_{pk2-pk11}\cdot\left[ \mathrm{PleC}_{kin2} \right]- k_{pk2-pk1}\cdot\left[ \mathrm{PleC}_{kin2} \right]+k_{pk1-pk2}\cdot\left[ \mathrm{PleC}_{kin1} \right]\cdot\left[ \mathrm{DivK} \right]- k_{pk2-pk3}\cdot\left[ \mathrm{PleC}_{kin2} \right]+ k_{pk3-pk2}\cdot\left[ \mathrm{PleC}_{kin3} \right]\cdot\left[ DivK\sim P \right]+ k_{pt2-pk2}\cdot\left[ \mathrm{PleC}_{pt2} \right]- k_{pk2-pt2}\cdot\left[ \mathrm{PleC}_{kin2} \right]$ | 10 |
| $\frac{d\left[ \mathrm{PleC}_{kin4} \right]}{dt} = k_{pk22-pk4}\cdot\left[ \mathrm{PleC}_{kin22} \right]-k_{pk4-pk22}\cdot\left[ \mathrm{PleC}_{kin4} \right]-k_{pk4-pk3}\cdot\left[ \mathrm{PleC}_{kin4} \right]+ k_{pk3-pk4}\cdot\left[ \mathrm{PleC}_{kin3} \right]\cdot\left[ \mathrm{DivK} \right] {+ k}_{pt4-pk4}\cdot\left[ \mathrm{PleC}_{pt4} \right]- k_{pk4-pt4}\cdot\left[ \mathrm{PleC}_{kin4} \right]$ | 11 |
| $\frac{d\left[ \mathrm{PleC}_{kin1} \right]}{dt} =-k_{pk1-pk0}\cdot\left[ \mathrm{Ple}C_{kin1} \right]\cdot\left[ DivK\sim P \right]+k_{pk0-pk1}\cdot\left[ \mathrm{Ple}C_{kin0} \right] -k_{pk1-pk2}\cdot\left[ \mathrm{Ple}C_{kin1} \right]\cdot\left[ \mathrm{DivK} \right]+k_{pk2-pk1}\cdot\left[ \mathrm{Ple}C_{kin2} \right]{+ k}_{pk2p-pk1} \cdot\left[ \mathrm{Ple}C_{kin2p} \right]\cdot\left[ DivK\sim P \right]- k_{pk1-pk2p}\cdot\left[ \mathrm{Ple}C_{kin1} \right]+ k_{pk1h-pk1}\cdot\left[ \mathrm{Ple}C_{kin1h} \right]- k_{pk1-pk1h}\cdot\left[ \mathrm{Ple}C_{kin1} \right]- k_{pk1-pk5}\cdot\left[ \mathrm{Ple}C_{kin1} \right]\cdot\left[ \mathrm{PleD} \right]+ k_{pk5-pk1}\cdot\left[ \mathrm{Ple}C_{kin5} \right]$ | 12 |
| $\frac{d\left[ \mathrm{PleC}_{kin3} \right]}{dt} =-k_{pk3-pk2}\cdot\left[ \mathrm{Ple}C_{kin3} \right]\cdot\left[ DivK\sim P \right]+k_{pk2-pk3}\cdot\left[ \mathrm{Ple}C_{kin2} \right] -k_{pk3-pk4}\cdot\left[ \mathrm{Ple}C_{kin3} \right]\cdot\left[ \mathrm{DivK} \right]+k_{pk4-pk3}\cdot\left[ \mathrm{Ple}C_{kin4} \right]{+ k}_{pk2p-pk3} \cdot\left[ \mathrm{Ple}C_{kin2p} \right]\cdot\left[ \mathrm{DivK} \right]- k_{pk3-pk2p}\cdot\left[ \mathrm{Ple}C_{kin3} \right]+ k_{pk3h-pk3}\cdot\left[ \mathrm{Ple}C_{kin3h} \right]-k_{pk3-pk3h}\cdot\left[ \mathrm{Ple}C_{kin3} \right]+ k_{pt3-pk3}\cdot\left[ \mathrm{PleC}_{pt3} \right]- k_{pk3-pt3}\cdot\left[ \mathrm{PleC}_{kin3} \right]- k_{pk3-pk6}\cdot\left[ \mathrm{Ple}C_{kin3} \right]\cdot\left[ \mathrm{PleD} \right]+ k_{pk6-pk3}\cdot\left[ \mathrm{Ple}C_{kin6} \right]$ | 13 |
| $\frac{d\left[ \mathrm{PleC}_{pt2} \right]}{dt} = k_{pk1h-pt2}\cdot\left[ \mathrm{PleC}_{kin1h} \right]\cdot\left[ DivK\sim P \right]- k_{pt2-pk1h}\cdot\left[ \mathrm{PleC}_{pt2} \right]- k_{pt2-pk2}\cdot\left[ \mathrm{PleC}_{pt2} \right]+ k_{pk2-pt2}\cdot\left[ \mathrm{PleC}_{kin2} \right]$ | 14 |
| $\frac{d\left[ \mathrm{PleC}_{pt3} \right]}{dt} = k_{pk1p-pt3}\cdot\left[ \mathrm{PleC}_{kin1p} \right]\cdot\left[ DivK\sim P \right]- k_{pt3-pk1p}\cdot\left[ \mathrm{PleC}_{pt3} \right]- k_{pt3-pk3}\cdot\left[ \mathrm{PleC}_{pt3} \right]+ k_{pk3-pt3}\cdot\left[ \mathrm{PleC}_{kin3} \right]$ | 15 |
| $\frac{d\left[ \mathrm{PleC}_{pt4} \right]}{dt} = k_{pk3h-pt4}\cdot\left[ \mathrm{PleC}_{kin3h} \right]\cdot\left[ DivK\sim P \right]- k_{pt4-pk3h}\cdot\left[ \mathrm{PleC}_{pt4} \right]- k_{pt4-pk4}\cdot\left[ \mathrm{PleC}_{pt4} \right]+ k_{pk4-pt4}\cdot\left[ \mathrm{PleC}_{kin4} \right] - k_{pt4-p11}\cdot\left[ \mathrm{PleC}_{pt4} \right]+ k_{p11-pt4}\cdot\left[ \mathrm{PleC}_{kin11} \right]$ | 16 |
| $\frac{d\left[ \mathrm{PleC}_{kin1h} \right]}{dt} = - k_{pk1h-pt2}\cdot\left[ \mathrm{PleC}_{kin1h} \right]\cdot\left[ DivK\sim P \right]+ k_{pt2-pk1h}\cdot\left[ \mathrm{PleC}_{pt2} \right]-k_{pk1h-pk1}\cdot\left[ \mathrm{Ple}C_{kin1h} \right]+ k_{pk1-pk1h}\cdot\left[ \mathrm{Ple}C_{kin1} \right]+ k_{pk1p-pk1h}\cdot\left[ \mathrm{Ple}C_{kin1p} \right]\cdot\left[ DivK\sim P \right]- k_{pk1h-pk1p}\cdot\left[ \mathrm{Ple}C_{kin1h} \right] - k_{pk1h-pt5}\cdot\left[ \mathrm{Ple}C_{kin1h} \right]\cdot\left[ PleD\sim P \right]+ k_{pt5-pk1h}\cdot\left[ \mathrm{Ple}C_{pt5} \right]$ | 17 |
| $\frac{d\left[ \mathrm{PleC}_{kin3h} \right]}{dt} = -k_{pk3h-pk3}\cdot\left[ \mathrm{Ple}C_{kin3h} \right]+ k_{pk3-pk3h}\cdot\left[ \mathrm{Ple}C_{kin3} \right]+ k_{pk1p-pk3h}\cdot\left[ \mathrm{Ple}C_{kin1p} \right]\cdot\left[ \mathrm{DivK} \right]- k_{pk3h-pk1p}\cdot\left[ \mathrm{Ple}C_{kin3h} \right]- k_{pk3h-pt4}\cdot\left[ \mathrm{PleC}_{kin3h} \right]\cdot\left[ DivK\sim P \right]+ k_{\mathrm{pt}4-pk3h}\cdot\left[ \mathrm{PleC}_{pt4} \right]- k_{pk3h-pt6}\cdot\left[ \mathrm{Ple}C_{kin3h} \right]\cdot\left[ PleD\sim P \right]+ k_{pt6-pk3h}\cdot\left[ \mathrm{Ple}C_{pt6} \right]$ | 18 |
| $\frac{d\left[ \mathrm{PleC}_{kin2p} \right]}{dt} = -k_{pk2p-pk1} \cdot\left[ \mathrm{Ple}C_{kin2p} \right]\cdot\left[ DivK\sim P \right]+ k_{pk1-pk2p}\cdot\left[ \mathrm{Ple}C_{kin1} \right] - k_{pk2p-pk3}\cdot\left[ \mathrm{Ple}C_{pk2p} \right]\cdot\left[ \mathrm{DivK} \right]+k_{pk3-pk2p}\cdot\left[ \mathrm{Ple}C_{kin3} \right] - k_{pk2p-pc}\cdot\left[ \mathrm{Ple}C_{kin2p} \right]$ | 19 |
| $\frac{d\left[ \mathrm{PleC}_{kin1p} \right]}{dt} = -k_{pk1p-pk1h} \cdot\left[ \mathrm{Ple}C_{kin1p} \right]\cdot\left[ DivK\sim P \right]+ k_{pk1h-pk1p}\cdot\left[ \mathrm{Ple}C_{kin1h} \right] - k_{pk1p-pt3}\cdot\left[ \mathrm{Ple}C_{pk1p} \right]\cdot\left[ \mathrm{DivK} \right]+k_{pt3-pk1p}\cdot\left[ \mathrm{Ple}C_{pt3} \right] - k_{pk1p-pc}\cdot\left[ \mathrm{Ple}C_{kin1p} \right]- k_{pk1p-pk3h}\cdot\left[ \mathrm{Ple}C_{kin1p} \right]\cdot\left[ \mathrm{DivK} \right]+ k_{pk3h-pk1p}\cdot\left[ \mathrm{Ple}C_{kin3h} \right]$ | 20 |
| $\frac{d\left[ \mathrm{DivJ} \right]}{dt} = -k_{j-jk}\cdot\left[ \mathrm{DivJ} \right]\cdot\left[ \mathrm{DivK} \right]+ k_{jk-j}\cdot\left[ DivJ:DivK \right]+ k_{e-jk}\cdot[DivJ:DivK]$ | 21 |
| $\frac{d\left[ DivJ:DivK \right]}{dt} = k_{j-jk}\cdot\left[ \mathrm{DivJ} \right]\cdot\left[ \mathrm{DivK} \right]- k_{jk-j}\cdot\left[ DivJ:DivK \right]- k_{e-jk}\cdot[DivJ:DivK]$ | 22 |
| $\frac{d\left[ \mathrm{PleD} \right]}{dt} = k_{syn-pld}- k_{deg-pld}\cdot\left[ \mathrm{PleD} \right]- k_{pk1-pk5}\cdot\left[ \mathrm{PleC}_{kin1} \right]\cdot\left[ \mathrm{PleD} \right]+k_{pk5-pk1}\cdot\left[ \mathrm{PleC}_{kin5} \right] - k_{pk3-pk6}\cdot\left[ \mathrm{PleC}_{kin3} \right]\cdot\left[ \mathrm{PleD} \right]+k_{pk6-pk3}\cdot\left[ \mathrm{PleC}_{kin6} \right] +k_{e-ph3}\cdot\left[ Phos:PleD\sim P \right]$ | 23 |
| $\frac{d\left[ PleD\sim P \right]}{dt} = - k_{deg-pldp}\cdot\left[ PleD\sim P \right]- k_{pk1h-pt5}\cdot\left[ \mathrm{Ple}C_{kin1h} \right]\cdot\left[ PleD\sim P \right]+ k_{pt5-pk1h}\cdot\left[ \mathrm{Ple}C_{pt5} \right]- k_{pk3h-pt6}\cdot\left[ \mathrm{Ple}C_{kin3h} \right]\cdot\left[ PleD\sim P \right]+ k_{pt6-pk3h}\cdot\left[ \mathrm{Ple}C_{pt6} \right]- k_{phos-ph3}\cdot\left[ \mathrm{Phos} \right]\cdot\left[ PleD\sim P \right]+ k_{ph3-phos}\cdot\left[ Phos:PleD\sim P \right]$ | 24 |
| $\frac{d\left[ \mathrm{PleC}_{kin5} \right]}{dt} = k_{pk1-pk5}\cdot\left[ \mathrm{PleC}_{kin1} \right]\cdot\left[ \mathrm{PleD} \right]-k_{pk5-pk1}\cdot\left[ \mathrm{PleC}_{kin5} \right]-k_{pk5-pt5}\cdot\left[ \mathrm{PleC}_{kin5} \right]+k_{pt5-pk5}\cdot\left[ \mathrm{PleC}_{pt5} \right]$ | 25 |
| $\frac{d\left[ \mathrm{PleC}_{kin6} \right]}{dt} = k_{pk3-pk6}\cdot\left[ \mathrm{PleC}_{kin3} \right]\cdot\left[ \mathrm{PleD} \right]-k_{pk6-pk3}\cdot\left[ \mathrm{PleC}_{kin6} \right]-k_{pk6-pt6}\cdot\left[ \mathrm{PleC}_{kin6} \right]+k_{pt6-pk6}\cdot\left[ \mathrm{PleC}_{pt6} \right]$ | 26 |
| $\frac{d\left[ \mathrm{PleC}_{pt5} \right]}{dt} = k_{pk5-pt5}\cdot\left[ \mathrm{PleC}_{kin5} \right]-k_{pt5-pk5}\cdot\left[ \mathrm{PleC}_{pt5} \right]{+ k}_{pk1h-pt5}\cdot\left[ \mathrm{Ple}C_{kin1h} \right]\cdot\left[ PleD\sim P \right]- k_{pt5-pk1h}\cdot\left[ \mathrm{Ple}C_{pt5} \right]$ | 27 |
| $\frac{d\left[ \mathrm{PleC}_{pt6} \right]}{dt} = k_{pk6-pt6}\cdot\left[ \mathrm{PleC}_{kin6} \right]-k_{pt6-pk6}\cdot\left[ \mathrm{PleC}_{pt6} \right]+ k_{pk3h-pt6}\cdot\left[ \mathrm{Ple}C_{kin3h} \right]\cdot\left[ PleD\sim P \right]- k_{pt6-pk3h}\cdot\left[ \mathrm{Ple}C_{pt6} \right]$ | 28 |
| $\frac{d\left[ \mathrm{Phos} \right]}{dt} =- k_{phos-ph3}\cdot\left[ \mathrm{Phos} \right]\cdot\left[ PleD\sim P \right]+ k_{ph3-phos}\cdot\left[ Phos:PleD\sim P \right] +k_{e-ph3}\cdot\left[ Phos:PleD\sim P \right]$ | 29 |
| $\frac{d\left[ Phos:PleD\sim P \right]}{dt} =k_{phos-ph3}\cdot\left[ \mathrm{Phos} \right]\cdot\left[ PleD\sim P \right]- k_{ph3-phos}\cdot\left[ Phos:PleD\sim P \right]-k_{e-ph3}\cdot\left[ Phos:PleD\sim P \right]$ | 30 |
| $\frac{d\left[ \mathrm{DivL} \right]}{dt} = k_{\mathrm{syndl}}-k_{\mathrm{degdl}}\cdot[DivL]-k_{dl-dldk}\cdot\left[ \mathrm{DivL} \right]\cdot\left[ DivK\sim P \right]+ k_{dldk-dl}\cdot\left[ DivL:DivK\sim P \right]$ | 31 |
| $\frac{d\left[ DivL:DivK\sim P \right]}{dt} = k_{dl-dldk}\cdot\left[ \mathrm{DivL} \right]\cdot\left[ DivK\sim P \right]- k_{dldk-dl}\cdot\left[ DivL:DivK\sim P \right]$ | 32 |
| $\frac{d\left[ \mathrm{CtrA} \right]}{dt} = k_{syn-ctr}- k_{deg-ctr}\cdot\left[ \mathrm{CtrA} \right]- k_{ck-ck1}\cdot\left[ \mathrm{CckA}_{\mathrm{kin}} \right]\cdot\left[ \mathrm{CtrA} \right]+k_{ck1-ck}\cdot\left[ \mathrm{CckA}_{kin1} \right] +k_{e-ch1}\cdot\left[ \mathrm{CckA}_{h1} \right]$ | 33 |
| $\frac{d\left[ CtrA\sim P \right]}{dt} = - k_{deg-ctr}\cdot\left[ CtrA\sim P \right]+ k_{ct1-ck}\cdot\left[ \mathrm{CckA}_{ct1} \right]-k_{ck-ct1}\cdot\left[ \mathrm{CckA}_{\mathrm{kin}} \right]\cdot\left[ CtrA\sim P \right] -k_{cp-ch1}\cdot\left[ \mathrm{CckA}_{\mathrm{phos}} \right]\cdot\left[ CtrA\sim P \right]+k_{ch1-cp}\cdot\left[ \mathrm{CckA}_{h1} \right]$ | 34 |
| $\frac{d\left[ \mathrm{CpdR} \right]}{dt} = k_{syn-cpd}- k_{deg-cpd}\cdot\left[ \mathrm{CpdR} \right]- k_{ck-ck2}\cdot\left[ \mathrm{CckA}_{\mathrm{kin}} \right]\cdot\left[ \mathrm{CpdR} \right]+k_{ck2-ck}\cdot\left[ \mathrm{CckA}_{kin2} \right] +k_{e-ch2}\cdot\left[ \mathrm{CckA}_{h2} \right]$ | 35 |
| $\frac{d\left[ CpdR\sim P \right]}{dt} = - k_{deg-cpd}\cdot\left[ CpdR\sim P \right]+ k_{ct2-ck}\cdot\left[ \mathrm{CckA}_{ct1} \right]-k_{ck-ct2}\cdot\left[ \mathrm{CckA}_{\mathrm{kin}} \right]\cdot\left[ CpdR\sim P \right] -k_{cp-ch2}\cdot\left[ \mathrm{CckA}_{\mathrm{phos}} \right]\cdot\left[ CpdR\sim P \right]+k_{ch2-cp}\cdot\left[ \mathrm{CckA}_{h2} \right]$ | 36 |
| $\frac{d\left[ \mathrm{CckA}_{\mathrm{phos}} \right]}{dt} = -k_{cp-ch1}\cdot\left[ \mathrm{CckA}_{\mathrm{phos}} \right]\cdot\left[ CtrA\sim P \right]+k_{ch1-cp}\cdot\left[ \mathrm{CckA}_{h1} \right] -k_{cp-ch2}\cdot\left[ \mathrm{CckA}_{\mathrm{phos}} \right]\cdot\left[ CpdR\sim P \right]+k_{ch2-cp}\cdot\left[ \mathrm{CckA}_{h2} \right] -k_{cp-ck}\cdot\left[ \mathrm{CckA}_{\mathrm{phos}} \right]\cdot\frac{\left[ \mathrm{DivL} \right]^{2}}{K_{\mathrm{dl}}^{2}+ \left[ \mathrm{DivL} \right]^{2}} +k_{ck-cp}\cdot\left[ \mathrm{CckA}_{\mathrm{kin}} \right]+k_{e-ch1}\cdot\left[ \mathrm{CckA}_{h1} \right]+k_{e-ch2}\cdot\left[ \mathrm{CckA}_{h2} \right]$ | 37 |
| $\frac{d\left[ \mathrm{CckA}_{\mathrm{kin}} \right]}{dt} = k_{cp-ck}\cdot\left[ \mathrm{CckA}_{\mathrm{phos}} \right]\cdot\frac{\left[ \mathrm{DivL} \right]^{2}}{K_{\mathrm{dl}}^{2} + \left[ \mathrm{DivL} \right]^{2}}-k_{ck-cp}\cdot\left[ \mathrm{CckA}_{\mathrm{kin}} \right] - k_{ck-ck1}\cdot\left[ \mathrm{CckA}_{\mathrm{kin}} \right]\cdot\left[ \mathrm{CtrA} \right]+k_{ck1-ck}\cdot\left[ \mathrm{CckA}_{kin1} \right]- k_{ck-ck2}\cdot\left[ \mathrm{CckA}_{\mathrm{kin}} \right]\cdot\left[ \mathrm{CpdR} \right]+k_{ck2-ck}\cdot\left[ \mathrm{CckA}_{kin2} \right]+ k_{ct1-ck}\cdot\left[ \mathrm{CckA}_{ct1} \right]-k_{ck-ct1}\cdot\left[ \mathrm{CckA}_{\mathrm{kin}} \right]\cdot\left[ CtrA\sim P \right] + k_{ct2-ck}\cdot\left[ \mathrm{CckA}_{ct2} \right]-k_{ck-ct2}\cdot\left[ \mathrm{CckA}_{\mathrm{kin}} \right]\cdot\left[ CpdR\sim P \right]$ | 38 |
| $\frac{d\left[ \mathrm{CckA}_{kin1} \right]}{dt} = k_{ck-ck1}\cdot\left[ \mathrm{CckA}_{\mathrm{kin}} \right]\cdot\left[ \mathrm{CtrA} \right]-k_{ck1-ck}\cdot\left[ \mathrm{CckA}_{kin1} \right] - k_{ck1-ct1}\cdot\left[ \mathrm{CckA}_{kin1} \right]+k_{ct1-ck1}\cdot\left[ \mathrm{CckA}_{ct1} \right]$ | 39 |
| $\frac{d\left[ \mathrm{CckA}_{kin2} \right]}{dt} = k_{ck-ck2}\cdot\left[ \mathrm{CckA}_{\mathrm{kin}} \right]\cdot\left[ \mathrm{CpdR} \right]-k_{ck2-ck}\cdot\left[ \mathrm{CckA}_{kin2} \right] - k_{ck2-ct2}\cdot\left[ \mathrm{CckA}_{kin2} \right]+k_{ct2-ck2}\cdot\left[ \mathrm{CckA}_{ct2} \right]$ | 40 |
| $\frac{d\left[ \mathrm{CckA}_{ct1} \right]}{dt} =k_{ck1-ct1}\cdot\left[ \mathrm{CckA}_{kin1} \right]-k_{ct1-ck1}\cdot\left[ \mathrm{CckA}_{ct1} \right]- k_{ct1-ck}\cdot\left[ \mathrm{CckA}_{ct1} \right]+k_{ck-ct1}\cdot\left[ \mathrm{CckA}_{\mathrm{kin}} \right]\cdot\left[ CtrA\sim P \right]$ | 41 |
| $\frac{d\left[ \mathrm{CckA}_{ct2} \right]}{dt} = k_{ck2-ct2}\cdot\left[ \mathrm{CckA}_{kin2} \right]-k_{ct2-ck2}\cdot\left[ \mathrm{CckA}_{ct2} \right]- k_{ct2-ck}\cdot\left[ \mathrm{CckA}_{ct2} \right]+k_{ck-ct2}\cdot\left[ \mathrm{CckA}_{\mathrm{kin}} \right]\cdot\left[ CpdR\sim P \right]$ | 42 |
| $\frac{d\left[ \mathrm{CckA}_{h1} \right]}{dt} = k_{cp-ch1}\cdot\left[ \mathrm{CckA}_{\mathrm{phos}} \right]\cdot\left[ CtrA\sim P \right]-k_{ch1-cp}\cdot\left[ \mathrm{CckA}_{h1} \right]-k_{e-ch1}\cdot\left[ \mathrm{CckA}_{h1} \right]$ | 43 |
| $\frac{d\left[ \mathrm{CckA}_{h2} \right]}{dt} = k_{cp-ch2}\cdot\left[ \mathrm{CckA}_{\mathrm{phos}} \right]\cdot\left[ CpdR\sim P \right]-k_{ch2-cp}\cdot\left[ \mathrm{CckA}_{h2} \right]-k_{e-ch2}\cdot\left[ \mathrm{CckA}_{h2} \right]$ | 44 |
