# Supplementary material for: Potential Role of a Bistable Histidine Kinase Switch in the Asymmetric Division Cycle of Caulobacter crescentus
Source: PLoS Comput Biol. 2013 Sep 12;9(9):e1003221. doi: 10.1371/journal.pcbi.1003221 (PMC3772055; doi:10.1371/journal.pcbi.1003221)
Supplement: Table S8 — Parameter values used to simulate mutants. (DOCX) [file pcbi.1003221.s014.docx]

| **Table S8:** Parameter values used to simulate mutants* | | | | | | | |
| --- | --- | --- | --- | --- | --- | --- | --- |
| *divK_D53N_* | | | | | | | |
| *k*_pk2-pt2_ | 0 | *k*_pt2-pk2_ | 0 | *k*_pk3-pt3_ | 0 | *k*_pt3-pk3_ | 0 |
| *k*_pk4-pt4_ | 0 | *k*_pt4-pk4_ | 0 | *k*_pt4-pk11_ | 0 | *k*_pk11-pt4_ | 0 |
| *divK_D90G_* | | | | | | | |
| *k*_ph1_pk11_ | 2.5 | *k*_pk11_ph1_ | 49 | *k*_ph1_pk12_ | 0.016 | *k*_pk12_ph1_ | 16 |
| *k*_ph2_pk22_ | 0.025 | *k*_pk22_ph2_ | 2.5e-03 | *k*_ph2_pk12_ | 1.6 | *k*_pk12_ph2_ | 16 |
| *k*_h1_h2_ | 0.016 | *k*_h2_h1_ | 1.6 | *k*_dl_dldk_ | 0.1 |  |  |
| *pleC:*Tn*5* | | | | | | | |
| PleC_tot | 0 |  |  |  |  |  |  |
| $\Delta divJ$ | | | | | | | |
| DivJ_tot | 0 |  |  |  |  |  |  |
| *pleC_F778L_* | | | | | | | |
| *k*_pk11-pk0_ | 0 | *k*_pk0-pk11_ | 0 | *k*_pk12-pk2_ | 0 | *k*_pk2-pk12_ | 0 |
| *k*_pk22-pk4_ | 0 | *k*_pk4-pk22_ | 0 |  |  |  |  |
| *Only parameter values deviating from wild type are listed. | | | | | | | |
