# Supplementary material for: Potential Role of a Bistable Histidine Kinase Switch in the Asymmetric Division Cycle of Caulobacter crescentus
Source: PLoS Comput Biol. 2013 Sep 12;9(9):e1003221. doi: 10.1371/journal.pcbi.1003221 (PMC3772055; doi:10.1371/journal.pcbi.1003221)
Supplement: Text S1 — Detailed description of model formulation. (DOCX) [file pcbi.1003221.s015.docx]

**Supplementary Material to:**

**Potential Role of a Bistable Histidine Kinase Switch in the Asymmetric Division Cycle of *Caulobacter crescentus***

Kartik Subramanian^1^, Mark R. Paul^2^ and John J. Tyson^3,4,*^

^1^Graduate Program in Genetics, Bioinformatics and Computational Biology, ^2^Department of Mechanical Engineering, ^3^Department of Biological Sciences, and ^4^Virginia Bioinformatics Institute, Virginia Polytechnic Institute and State University, Blacksburg VA 24061

In **Figure S1** we propose a set of molecular interactions governing the PleC phosphatase-to-kinase transition. Each step in the mechanism is an elementary chemical reaction governed by mass-action kinetics. To each species in the mechanism we assign a standard Gibbs free energy that is consistent with our estimate of the standard Gibbs free energy change for the reaction, $\Delta G^{0}$. To each reaction we then assign forward and reverse rate constants that are consistent with the equilibrium constant of the reaction, *K*_eq_ = $e^{-{\Delta G^{0}}/{RT}}$. In this way, we can be sure that our reaction mechanism satisfies the principle of detailed balance around every closed loop of chemical reactions.

In the following sections, we (A) review some basic concepts of histidine kinases and allosteric enzymes, (B) assign free energies to the chemical species and estimate the standard free energy change for all the reactions in **Figure S1**, (C) explain our rationale for assigning rate constants to all reactions, and (D) propose a simplified reaction mechanism that retains the crucial feature of bistability.

A. Histidine kinases and allosteric enzymes

*Histidine kinases in two-component systems*

A common signaling motif in microbes is the ‘two-component system’ comprised of a histidine kinase (HK) and a response regulator (RR). The HK component, typically a homodimer located in the bacterial membrane, responds to environmental stimuli by autophosphorylating a histidine residue and then transfering the phosphoryl group to an aspartate residue of the RR. HKs are bifunctional, in that they can serve as either a kinase or a phosphatase to a given RR. In keeping with this complexity, HKs have an elaborate and modular design. Each monomer has three subdomains: a membrane-bound sensor domain, a dimerization domain containing the phospho-accepting His residue, and a catalytic domain containing an ATP binding site [1]. It is believed that an HK, in response to sensory input via its sensor domain, changes its conformation to act as either a kinase or a phosphatase. This view of two-component systems is a generalization, and no two are alike. There are variations of the general scheme both in terms of architecture and activity. Some two-component systems have more than two components, forming a chain of alternating His and Asp containing domains [2]. Each domain may be on a distinct protein, or some of the domains may be parts of a single protein [3]. The exact details of how the change between kinase and phosphatase forms is achieved also vary, and different mechanisms have been suggested [1].

PleC, an HK in *Caulobacter*, has its own unique traits. Although it is bifunctional, its function is not regulated by ligand binding to a sensor domain. Instead, its RR (DivK) up-regulates the kinase form of PleC [4]. DivK binds to only one site on PleC [5] and, interestingly, PleC’s sensor domain is not essential to its role in cell cycle progression [6]. These observations suggest that, when DivK binds to PleC, it acts as substrate/product and allosteric modulator concurrently. These dual roles of DivK (and DivK~P) are central to our model (**Figure S1**).

*MWC model of allosteric regulation*

Allosteric regulation can be explained using either the Monod-Wyman-Changeux (MWC) model [7] or the Koshland-Némethy-Filmer (KNF) model [8]. The main difference between the two is that KNF allows monomers within a single protein complex to exist in different states while MWC does not. As a result, the MWC ‘dose-response’ curve is more sigmoidal, and hence in our opinion, more suited to explain the switch-like transition from swarmer cell to stalked cell in the *Caulobacter* cell cycle. **Figure S2** illustrates the general idea behind the MWC model. The allosteric protein can exist in either an inactive form (R for ‘relaxed’) or an active form (T for ‘tensed’). In the absence of ligand binding, the R form is more stable than the T form. However, ligand binding stabilizes the T form to a greater extent than it does the R form (i.e., the TL intermediate has a lower free energy than the RL intermediate). In short, in the absence of ligand, equilibrium favors an excess of R over T, while in the presence of the ligand, the TL form is in excess [9].

B. Thermodynamic considerations

*Free energy change of a chemical reaction and definition of ‘standard state’*

The free energy change of a chemical reaction, A + B 🡪 C + D, is

$$\Delta G=\Delta G^{0}+RT\text{ln}\left( \frac{a_{\text{C}}a_{\text{D}}}{a_{\text{A}}a_{\text{B}}} \right)$$

where Δ*G*^0^ is the standard free energy change of the reaction, and *a*_A_ etc. are the ‘activities’ of the reactants and products. For an ideal solution (a reasonable approximation for our purposes), *a*_A_ = [A]/[A]^0^, where [A]^0^ is the ‘standard’ concentration of species A. (Clearly, Δ*G*^0^ is the free energy change of the reaction when all participating species are present at their standard concentrations; i.e., *a*_A_ = 1, etc.) In physical chemistry textbooks, it is conventional to define the standard concentration of all chemical species to be 1 M, but this definition is neither necessary nor desirable. Even physical chemists define the standard concentration of H_2_O to be 55.5 M (the unchanging concentration of water in dilute aqueous solutions), and biochemists find it convenient to define the standard concentration of H^+^ to be 10^−7^ M (i.e., neutral pH, characteristic of living cells). We will define the standard states of the chemical species in our reaction mechanism to make thermodynamic calculations and kinetic simulations as simple as possible.

The kinetic rate constants for a chemical reaction are related to Δ*G*^0^ for the reaction through the equilibrium constant, *K*_eq_ = $e^{-{\Delta G^{0}}/{RT}}$, where *R* = gas constant and *T* = absolute temperature (*RT* = 2.48 kJ/mole at 298 K) and *K*_eq_ is the mass-action quotient at equilibrium:

$$K_{\text{eq}}=\frac{a_{\text{C,eq}}a_{\text{D,eq}}}{a_{\text{A,eq}}a_{\text{B,eq}}}$$

For an elementary chemical reaction (described by mass-action kinetics), the rates of the forward and reverse reactions are

$k_{\text{f}}\cdot a_{\text{A}}\cdot a_{\text{B}}, k_{\text{r}}\cdot a_{\text{C}}\cdot a_{\text{D}}$,

respectively. At equilibrium, the rates of the forward and reverse reactions are equal; hence, *K*_eq_ = *k*_f_/*k*_r_.

For numerical simulations of a chemical reaction system, it is convenient to scale the concentrations of all time-dependent intermediates so that *a*_A_(*t*) ≈ 1, etc. Hence, to connect our thermodynamics estimates of Δ*G*^0^ and *K*_eq_ to our kinetic simulations, we must choose the ‘standard’ concentrations of our species to be the typical intracellular concentrations used to scale the kinetic rate equations.

As an example of these ideas, consider the hydrolysis of ATP in *Caulobacter* cells. It is reasonable to assume that the concentrations of ATP, ADP and P_i_ are constant in the cell (say, [ATP] = 13 mM, [ADP] = 2 mM, [P_i_] = 2 mM). We take these intracellular concentrations as our ‘standard’ concentrations for ATP, ADP and Pi, so that *a*_ATP_ = *a*_ADP_ = *a*_Pi_ = 1 at all times. With this choice, the concentrations of ATP, ADP and P_i_ drop out of all our equilibrium calculations and kinetic rate laws, because their activities are always ≡ 1 (in the same way that [H_2_O] drops out of all such calculations because the standard concentration of water is defined to be 55.5 M). With this definition of standard state, we have

ATP + H_2_O 🡪 ADP + P_i_, Δ*G*^0^ = −50 kJ/mole

for ATP hydrolysis under these (standard) conditions inside the cell.

*Assigning standard free energy changes to the generic reactions in our model*

In **Table S2** we catalog the generic reactions in our model (**Figure S1**), starting with the phosphorylation of ADP to ATP (reaction R1).

To estimate Δ*G*^0^ for the auto-phosphorylation of PleC, we observe that HKs auto-phosphorylate on a histidine residue, resulting in a phosphoramidate bond. Unlike phosphorylation on serine, threonine or tyrosine residues that give rise to stable phosphoesters, phosphoramidates are high-energy bonds [10,11]. As a result, HK auto-phosphorylation may have $\Delta G^{0}\approx0.$ To estimate Δ*G*^0^ for PleC auto-phosphorylation (for which there are no data), we refer to the case of CheA, a more well studied HK. In the presence of ATP alone, CheA auto-phosphorylation is reported to be highly endergonic (Δ*G*^0^ = +12.3 kJ/mole [12] and Δ*G*^0^  = + 4.8 kJ/mole [13].) However, in the presence of CheW and Tar receptors, CheA shows a 300-fold increase in kinase activity [14] and a 10- to 100-fold increase in autokinase activity [15]. Hence, under favorable conditions, CheA auto-phosphorylation is close to equilibrium, if not exergonic. Therefore, it is reasonable to assume that, when bound to DivK or DivK~P, PleC auto-phosphorylation is close to equilibrium under typical intracellular conditions:

PleC_kin_ + ATP 🡪 PleC_kin_~P + ADP, Δ*G*^0^ = 0, *K*_eq_ = 1

From this assumption and Δ*G*^0^ for ATP hydrolysis, we calculate Δ*G*^0^ for the phosphorylation of PleC_kin_ (reaction R2 in **Table S2**). Loosely speaking, we may say that PleC_kin_~P is 50 kJ/mole higher in Gibbs free energy than PleC_kin_.

HK~P binds to its cognate RR and transfers its phosphate group to an aspartate residue on the RR. The resultant acyl phosphate (RR-Asp~P) is also a high-energy bond, suggesting that the phospho-transfer reaction is reversible [11]. However, RR-Asp~P is thought to undergo a conformational change that makes it more stable [16], so that the free energy of hydrolysis of RR~P is not as negative as that of ATP (−50 kJ/mole) (**Figure S3A**). Here again, in the absence of any data on the free energy of hydrolysis of DivK~P, we assume that

DivK~P + H_2_O 🡪 DivK + P_i_, Δ*G*^0^ = −30 kJ/mole

under (standard) conditions inside the cell. Loosely speaking, we may say that DivK~P is 30 kJ/mole higher in free energy than DivK (reaction R3 in **Table S2**).

Next, consider the R_0_ 🡪 T_0_ reaction in our MWC representation of the PleC kinase-to-phosphatase transition. Given that PleC is a phosphatase by default and requires DivK to become a kinase, we choose PleC phosphatase to be the R form and PleC kinase to be the T form. We assume PleC_kin_ is higher in free energy by 8.55 kJ/mole (reaction R4 in **Table S2**). Taking into account that PleC is a dimer, we have, for the phosphatase-to-kinase transition in the absence of DivK,

(PleC_pho_)_2_ 🡪 (PleC_kin_)_2_, Δ*G*^0^ = 17.1 kJ/mole, *K*_eq_ = 10^−3^.

There is a change in free energy with the binding of successive molecules of DivK to each PleC monomer (reactions R5-R8 in **Table S2**). We propose that each Δ*G*^0^ should be a sum of the individual changes associated with DivK’s role as a substrate or product and as an allosteric ligand. **Table S3** lists the Δ*G*^0^ values we assume for each effect. Formation of an enzyme-substrate complex, being a favorable reaction, has been assigned Δ*G*^0^ $\leq$ 0. Enzyme-product complexes presumably dissociate, and hence have been assigned Δ*G*^0^ > 0. The free energy change when ligand binds to the R form (PleC_pho_) has to be smaller than when it binds to the T form (PleC_kin_). For the sake of simplicity, we set the drop in free energy associated with formation of RL_1_ and RL_2_ complexes to be 0. For the formation of TL_1_ and TL_2_ complexes, we assume that $\Delta G_{\mathrm{KL}}^{0}$ = −14.25 kJ/mole. Based on the Δ*G*^0^ values in **Table S3**, we assign free energy changes for the typical ligand binding reactions (R5-R8) in **Table S2**.

Next, we use R5-R8 to calculate Δ*G*^0^ values for all the binding reactions in our wiring diagram. For example, to calculate Δ*G*^0^ for the reaction (PleC_pho_:DivK)_2_ 🡪 (PleC_kin_:DivK)_2_, we notice that this reaction is equivalent to 2(R4 + R8 − R6), so Δ*G*^0^ for the overall reaction is 2(8.55 – 19.95 − 11.4) = −45.6 kJ/mole. Similarly, we express DivK~P:PleC_pho_:PleC_pho_:DivK 🡪 DivK~P:PleC_kin_:PleC_kin_:DivK as the sum −R5 − R6 + 2R4 + R7 + R8, so Δ*G*^0^ for the overall reaction is 0 – 11.4 + 17.1 – 8.55 – 19.95 = −22.8 kJ/mole. In like manner, Δ*G*^0^ for the reaction (PleC_pho_:DivK~P)_2_ 🡪 (PleC_kin_:DivK~P)_2_ is 0. All other binding reactions are treated the same way.

To make these Δ*G*^0^ calculations easier, we can assign ‘baseline’ free energies to species ($\text{∆}\text{G}_{\text{A}}^{\text{\#}}$) such that $\text{∆}\text{G}^{\text{0}}\text{=∆}\text{G}_{\text{C}}^{\text{\#}}\text{+∆}\text{G}_{\text{D}}^{\text{\#}}\text{-∆}\text{G}_{\text{A}}^{\text{\#}}\text{-∆}\text{G}_{\text{B}}^{\text{\#}}$. For example, if we assign a baseline free energy of 0 to (PleC_pho_)_2_ and to DivK, then (see **Figure S1**)

Δ*G*^#^ = 17.1 kJ/mole for (PleC_kin_)_2_

Δ*G*^#^ = 30 kJ/mole for DivK~P

Δ*G*^#^ = 11.4 kJ/mole for PleC_pho_:PleC_pho_:DivK

Δ*G*^#^ = 30 kJ/mole for PleC_pho_:PleC_pho_:DivK~P

Δ*G*^#^ = 41.4 kJ/mole for DivK:PleC_pho_:PleC_pho_:DivK~P

From these free energies, we can calculate Δ*G*^0^ for the phosphatase reaction (R9)

PleC_pho_:PleC_pho_:DivK~P + H_2_O 🡪 PleC_pho_:PleC_pho_:DivK + P_i_,

Δ*G*^0^ = −18.6 kJ/mole, *K*_eq_ = 1.8×10^3^.

In like manner, we assign baseline free energies to all the species in **Figure 1**.

To estimate Δ*G*^0^ for phosphotransfer from PleC_kin_~P to DivK (R10 in **Table S2**), we consider the reaction cycle in **Figure S3B**. The net reaction of this cycle is

DivK + ATP 🡪 DivK~P + ADP, Δ*G*^0^ = −20 kJ/mole, *K*_eq_ = 3.2×10^3^.

At equilibrium (recalling that *a*_ATP_ = *a*_ADP_ = 1):

$$K_{\mathrm{eq}}=\frac{k_{pk3-pk4}}{k_{pk4-pk3}} \cdot\frac{k_{pk4-pt4}}{k_{pt4-pk4}} \cdot\frac{k_{pt4-pk3h}}{k_{pk3h-pt4}} \cdot\frac{k_{pk3h-pk3}}{k_{pk3-pk3h}}=\frac{\left[ DivK\sim P \right]}{\left[ \mathrm{DivK} \right]}=e^{20/2.48}\approx{3.2\times10}^{3}$$

Using *K*_eq_ values from **Table S2**,

${3.2\times10}^{3}=(3162) \cdot\frac{k_{\text{pt4-pk3h}}}{k_{\text{pk3h-pt4}}} \cdot(\frac{1}{31.62})\cdot(1)$,

we calculate the equilibrium constant for the phosphotransfer reaction to be

PleC_kin_~P:DivK 🡪 PleC_kin_:DivK~P, $K_{\mathrm{pt}}=\frac{k_{\text{pt4-pk3h}}}{k_{\text{pk3h-pt4}}}=32$, ${\text{∆}\text{G}}_{\text{pt}}^{\text{0}}\text{=}\text{ }\text{-8.6 kJ/mole}$.

Finally, HK molecules undergo auto-dephosphorylation (R11 in **Table S2**) in which the high-energy phosphoryl group (+50 kJ/mole) is hydrolyzed to give inorganic phosphate. In our model we set this to be an irreversible reaction because of the large free energy change accompanying the reaction.

We assume that PleD and PleD~P have similar baseline free energies as DivK and DivK~ P, respectively. Hence, the equilibrium constant for the phosphotransfer reaction is the same for PleD as for DivK:

Δ*G*^#^ = 0 kJ/mole for PleD

Δ*G*^#^ = 30 kJ/mole for PleD~P

PleC_kin_~P:PleD 🡪 PleC_kin_:PleD~P, $K_{\mathrm{pt}}=32$, ${\text{∆}\text{G}}_{\text{pt}}^{\text{0}}\text{=}\text{ }\text{-8.6 kJ/mole}$.

C. Kinetic parameters used in the model.

The choice of parameter values is constrained by the fact that the ratio of the forward and reverse rate constants for any reaction is equal to its computed equilibrium constant. As a first approximation, for the main part of **Figure S1**, we set the rate constant in the spontaneous direction to be 5 min^-1^, and then set the rate constant in the reverse direction so that the ratio of rate constants = *K*_eq_. This simple choice immediately generated bistability in the output of the HK switch (see **Figure 3** in the main text)

In order to get sufficient phosphorylation of PleD by PleC_kin_, we made the PleD-associated rate constants 2 fold faster than the DivK-associated rate constants (see the lower part of **Figure S1**).

**Table S4** and **S5** lists the equations and parameters used in the model.

D. Model reduction

Our model of the DivJ-PleC-DivK system is comprised of 30 differential equations and 83 rate constants (**Table S4** Eq.1-30). It contains necessary details regarding the three roles of PleC histidine kinase in autophosphorylation, phosphotransfer and phosphatase reactions. It incorporates a general understanding of enzyme-substrate reactions, allosteric theory and detailed balance, in order to formulate a reaction scheme that is consistent with both *in vivo* and *in vitro* experimental observations. Ultimately though, we admit that the full model is unwieldy, especially if it has to be integrated into a larger model describing other components of the gene/protein regulatory network controlling the asymmetric division cycle of *Caulobacter*. Therefore, we propose a reduced model (**Figure S4**), based on the following reasonable assumptions. In **Figure S5** we plot one-parameter bifurcation diagrams for the reduced model in order to show that its behavior is comparable to the full model.

Assumption 1. We have eliminated those components of the full model (namely, PleC_kin_, PleC_pt3h_ and PleC_kin_:DivK) that are present at concentrations below 0.01.

Assumption 2. We assumed that other components (PleC_ph1p_ and PleC_ph2p_) are always at steady state values:

$\left[ \mathrm{PleC}_{ph1p} \right]_{\mathrm{ss}} =\frac{k_{pk1p-ph1p}\cdot\left[ \mathrm{Ple}C_{kin1p} \right]}{k_{ph1p-pc}+k_{ph1p-pk1p}}$ and $\left[ \mathrm{PleC}_{ph2p} \right]_{\mathrm{ss}} =\frac{k_{pk2p-h2p}\cdot\left[ \mathrm{Ple}C_{kin1p} \right]}{k_{ph2p-pc}+k_{ph2p-pk2p}}$

Substituting these relationships into Eqs. 21 and 22 of **Table S4** gives the following modified equations and parameters (see also **Tables S6** and **S7**):

| $\frac{d\left[ \mathrm{PleC}_{kin1p} \right]}{dt} = - k_{pk1p-pc}\cdot\left[ \mathrm{Ple}C_{kin1p} \right]+ other terms$ | $k_{pk1p-pc} =\frac{k_{pk1p-ph1p}+ k_{ph1p-pc}}{k_{ph1p-pk1p}}$ |
| --- | --- |
|  |  |
| $\frac{d\left[ \mathrm{PleC}_{kin2p} \right]}{dt} = - k_{pk2p-pc}\cdot\left[ \mathrm{Ple}C_{kin2p} \right]+other terms$ | $k_{pk2p-pc} =\frac{k_{pk2p-ph2p}+ k_{ph2p-pc}}{k_{ph2p-pk2p}}$ |

Assumption 3. The reaction $\mathrm{PleC}_{ph11} \leftrightarrow\mathrm{PleC}_{kin11}$ is at equilibrium, so we combined the two variables into a single modified variable PleC_kin11_. The modified differential equation and parameters for PleC_kin11_ is given in **Table S6** and **S7**.

Assumption 4. PleC_ph22_ was also assumed to be at steady state:

$$\left[ \mathrm{PleC}_{ph22} \right]_{\mathrm{ss}} =\frac{k_{ph2-ph22}\cdot\left[ \mathrm{Ple}C_{ph2} \right]\cdot\left[ \mathrm{DivK} \right]+ k_{pk22-ph22}\cdot\left[ \mathrm{Ple}C_{kin22} \right]}{k_{ph22-ph2}+k_{ph22-pk22}}$$

Substituting this expression into Equations 2, 5 & 11 of **Table S4** gives the following modified equations and parameters (see also **Table S6** and **S7**):

| $\frac{d\left[ \mathrm{PleC}_{ph2} \right]}{dt} = -k_{ph2-pk22}\cdot\left[ \mathrm{Ple}C_{ph2} \right]\cdot\left[ \mathrm{DivK} \right]+ k_{pk22-ph2}\cdot\left[ \mathrm{PleC}_{kin22} \right] +other terms$ | |
| --- | --- |
| $\frac{d\left[ \mathrm{PleC}_{kin22} \right]}{dt} = k_{ph2-pk22}\cdot\left[ \mathrm{Ple}C_{ph2} \right]\cdot\left[ \mathrm{DivK} \right]- k_{pk22-ph2}\cdot\left[ \mathrm{PleC}_{kin22} \right] +other terms$ | |
| $\frac{d\left[ \mathrm{DivK} \right]}{dt} = -k_{ph2-pk22}\cdot\left[ \mathrm{Ple}C_{ph2} \right]\cdot\left[ \mathrm{DivK} \right]+ k_{pk22-ph2}\cdot\left[ \mathrm{PleC}_{kin22} \right] +other terms$ | |
| $k_{ph2-pk22} =\frac{k_{ph2-ph22}\cdot k_{ph22-pk22}}{k_{ph22-ph2}+k_{ph22-pk22}}$ | $k_{pk22-ph2} =\frac{k_{pk22-ph22}\cdot k_{ph22-ph2}}{k_{ph22-ph2}+k_{ph22-pk22}}$ |

Assumption 5. In a similar fashion, we made a steady-state approximation for PleC_ph12_:

| $\left[ \mathrm{PleC}_{ph12} \right]_{\mathrm{ss}}=\frac{k_{ph2-ph12}\cdot\left[ \mathrm{Ple}C_{ph2} \right]\cdot\left[ DivK\sim P \right]+ k_{ph1-ph12}\cdot\left[ \mathrm{Ple}C_{ph1} \right]\cdot\left[ \mathrm{DivK} \right]+k_{pk12-ph12}\cdot\left[ \mathrm{Ple}C_{kin22} \right]}{k_{ph12-ph2}+k_{ph12-pk12} +k_{ph12-ph2}}$ | |
| --- | --- |
| $k_{ph1-pk12} =\frac{k_{ph1-ph12}\cdot k_{ph12-pk12}}{k_{ph12-ph1}+k_{ph12-ph2}+k_{ph12-pk12}}$ | $k_{pk12-ph1} =\frac{k_{pk12-ph12}\cdot k_{ph12-ph1}}{k_{ph12-ph1}+k_{ph12-ph2}+k_{ph12-pk12}}$ |
| $k_{ph2-pk12} =\frac{k_{ph2-ph12}\cdot k_{ph12-pk12}}{k_{ph12-ph1}+k_{ph12-ph2}+k_{ph12-pk12}}$ | $k_{pk12-pk12} =\frac{k_{pk12-ph12}\cdot k_{ph12-ph2}}{k_{ph12-ph1}+k_{ph12-ph2}+k_{ph12-pk12}}$ |
| $k_{h1-h2} =\frac{k_{ph1-ph12}\cdot k_{ph12-ph2}}{k_{ph12-ph1}+k_{ph12-ph2}+k_{ph12-pk12}}$ | $k_{h2-h1} =\frac{k_{ph2-ph12}\cdot k_{ph12-ph1}}{k_{ph12-ph1}+k_{ph12-ph2}+k_{ph12-pk12}}$ |

These changes propagate to the differential equations for PleC_kin12_, PleC_ph2_, PleC_ph1_, DivK and DivK~P, as documented in **Table S6**.

**References:**

1. Stewart RC (2010) Protein histidine kinases: assembly of active sites and their regulation in signaling pathways. Current Opinion in Microbiology 13: 133–141. Available: http://www.pubmedcentral.nih.gov/articlerender.fcgi?artid=2847664&tool=pmcentrez&rendertype=abstract.

2. Perego M, Hoch JA (1996) Protein aspartate phosphatases control the output of two-component signal transduction systems. Trends in Genetics 12: 97–101. Available: http://www.ncbi.nlm.nih.gov/pubmed/8868347.

3. Georgellis D, Kwon O, De Wulf P, Lin EC (1998) Signal decay through a reverse phosphorelay in the Arc two-component signal transduction system. The Journal of Biological Chemistry 273: 32864–32869. Available: http://www.ncbi.nlm.nih.gov/pubmed/9830034.

4. Paul R, Jaeger T, Abel S, Wiederkehr I, Folcher M, et al. (2008) Allosteric regulation of histidine kinases by their cognate response regulator determines cell fate. Cell 133: 452–461. Available: http://www.pubmedcentral.nih.gov/articlerender.fcgi?artid=2804905&tool=pmcentrez&rendertype=abstract.

5. Ohta N, Newton A (2003) The Core Dimerization Domains of Histidine Kinases Contain Recognition Specificity for the Cognate Response Regulator. Journal of bacteriology 185: 4424–4431. Available: http://jb.asm.org/content/185/15/4424.abstract

6. Wang SP, Sharma PL, Schoenlein P V, Ely B (1993) A histidine protein kinase is involved in polar organelle development in Caulobacter crescentus. Proceedings of the National Academy of Sciences of the United States of America 90: 630–634. Available: http://www.pubmedcentral.nih.gov/articlerender.fcgi?artid=45717&tool=pmcentrez&rendertype=abstract.

7. Rubin MM, Changeux JP (1966) On the nature of allosteric transitions: implications of non-exclusive ligand binding. Journal of Molecular Biology 21: 265–274. Available: http://www.ncbi.nlm.nih.gov/pubmed/5972463.

8. Koshland DE Jr, Nemethy G, FIlmer D: Comparison of experimental binding data and theoretical models in protein containing subunits. (1965) Biochemistry 5: 365–385. Available: http://pubs.acs.org/doi/abs/10.1021/bi00865a047

9. Changeux J-P (2011) Allostery and the Monod-Wyman-Changeux Model After 50 Years. Annual review of biophysics 41: 103–133. Available: http://www.ncbi.nlm.nih.gov/pubmed/22224598.

10. Stock AM, Robinson VL, Goudreau PN (2000) Two-component signal transduction. Annual Review of Biochemistry 69: 183–215. Available: http://www.ncbi.nlm.nih.gov/pubmed/10966457.

11. Perry J, Koteva K, Wright G (2011) Receptor domains of two-component signal transduction systems. Molecular Biosystems 7: 1388–1398. Available: http://www.ncbi.nlm.nih.gov/pubmed/21347487.

12. Wylie D, Stock A, Wong CY, Stock J (1988) Sensory transduction in bacterial chemotaxis involves phosphotransfer between Che proteins. Biochemical and Biophysical Research Communications 151: 891–896. Available: http://linkinghub.elsevier.com/retrieve/pii/S0006291X88803656.

13. Tawa P, Stewart RC (1994) Kinetics of CheA autophosphorylation and dephosphorylation reactions. Biochemistry 33: 7917–7924. Available: http://www.ncbi.nlm.nih.gov/pubmed/8011654.

14. Borkovich KA, Kaplan N, Hess JF, Simon MI (1989) Transmembrane signal transduction in bacterial chemotaxis involves ligand-dependent activation of phosphate group transfer. Proceedings of the National Academy of Sciences of the United States of America 86: 1208–1212. Available: http://www.pubmedcentral.nih.gov/articlerender.fcgi?artid=286655&tool=pmcentrez&rendertype=abstract.

15. Levit MN, Liu Y, Stock JB (1999) Mechanism of CheA protein kinase activation in receptor signaling complexes. Biochemistry 38: 6651–6658. Available: http://www.ncbi.nlm.nih.gov/pubmed/10350484.

16. Stock J, Da Re S (2000) Signal transduction: response regulators on and off. Current Biology 10: R420–R424. Available: http://www.ncbi.nlm.nih.gov/pubmed/10837243
